# The adaptor molecule CD2AP in CD4 T cells modulates differentiation of follicular helper T cells during chronic LCMV infection

**DOI:** 10.1371/journal.ppat.1007053

**Published:** 2018-05-07

**Authors:** Saravanan Raju, Kohei Kometani, Tomohiro Kurosaki, Andrey S. Shaw, Takeshi Egawa

**Affiliations:** 1 Department of Pathology and Immunology, Washington University School of Medicine, Saint Louis, Missouri, United States of America; 2 Laboratory for Lymphocyte Differentiation, RIKEN Center for Integrative Medical Sciences, Yokohama, Kanagawa, Japan; 3 Laboratory of Lymphocyte Differentiation, WPI Immunology Frontier Research Center, Osaka University, Osaka, Japan; University of Rochester Medical Center, UNITED STATES

## Abstract

CD4 T cell-mediated help to CD8 T cells and B cells is a critical arm of the adaptive immune system required for control of pathogen infection. CD4 T cells express cytokines and co-stimulatory molecules that support a sustained CD8 T cell response and also enhance generation of protective antibody by germinal center B cells. However, the molecular components that modulate CD4 T cell functions in response to viral infection or vaccine are incompletely understood. Here we demonstrate that inactivation of the signaling adaptor CD2-associated protein (CD2AP) promotes CD4 T cell differentiation towards the follicular helper lineage, leading to enhanced control of viral infection by augmented germinal center response in chronic lymphocytic choriomeningitis virus (LCMV) infection. The enhanced follicular helper differentiation is associated with extended duration of TCR signaling and enhanced cytokine production of CD2AP-deficient CD4 T cells specifically under T_H_1 conditions, while neither prolonged TCR signaling nor enhanced follicular helper differentiation was observed under conditions that induce other helper effector subsets. Despite the structural similarity between CD2AP and the closely related adaptor protein CIN85, we observed defective antibody-mediated control of chronic LCMV infection in mice lacking CIN85 in T cells, suggesting non-overlapping and potentially antagonistic roles for CD2AP and CIN85. These results suggest that tuning of TCR signaling by targeting CD2AP improves protective antibody responses in viral infection.

## Author summary

Enhancing the production of protective antibodies in response to infection or vaccine is critically important for host protection. However, we have only limited knowledge about molecular targets to enhance functions of CD4 helper T cells that are essential for antibody affinity maturation and class switching. In this work, we found that inhibiting the function of the adaptor molecule CD2AP results in enhanced antibody responses and improved protection of mice from chronic infection by LCMV. Mice lacking CD2AP specifically in T cells showed enhanced CD4 T cell differentiation towards the follicular helper subset, which is a critical regulator of antibody responses, and generated more germinal center B cells leading to production of mutated, protective antibodies. This effect was specific to CD4 T cells in type-I immune responses, associated with viral infection, while deletion of CD2AP had little impact on CD4 T cells in type-II immune responses or CD8 T cells. Our results thus suggest that CD2AP can be a specific target to enhance antiviral protective immunity during viral infection or vaccination.

## Introduction

CD4 T lymphocytes are critical mediators of the adaptive immune response to infection [1]. Upon encountering cognate antigen as peptide-MHC (pMHC) complexes on antigen presenting cells in the lymphoid organs, they initiate clonal expansion and undergo differentiation into effector cells, depending on the cytokine milieu initially established by innate immune cells [2]. In response to viral infection, differentiation of activated CD4 T cells is directed towards an IFN-γ-producing subset, referred to as T_H_1, that also produces IL-2 and TNF-α and enhances cellular immune responses by CD8 T cells and macrophages. Activated CD4 T cells also differentiate into the follicular helper (T_FH_) cells that migrate to germinal centers (GCs) [3]. During viral infection, T_FH_ cells provide help signals to B cells for antibody affinity maturation through their expression of co-stimulatory ligands and cytokines, such as CD40L and IL-21, and direct immunoglobulin class switching to the antiviral IgG2a/c subclass by secreting IFN-γ [4]. Thus, understanding the molecular mechanisms governing CD4 T cell responses through either TCR-dependent or cytokine-dependent signals can yield insight into host protection against viral infection or protective antibodies induced by vaccines.

Following ligation of the TCR by cognate pMHC complexes, re-organization of membrane proteins and post-translational modification of signaling components ultimately result in activation of genes necessary for their proliferation and effector differentiation [5]. At the TCR-juxta-membrane regions, the TCR itself and its proximal kinases are densely packed and surrounded by adhesion molecules, such as LFA1 integrin, which together comprise the immunological synapse (IS) and facilitate quality control of TCR signals [6]. A number of previous studies have demonstrated that repeated cycles of this brief activation of TCR fine-tune the quality and magnitude of the activation program [7]. However, it remains unknown whether such fine-tuning mechanisms are common across different effector subsets, or whether the altered regulation may have impact on CD4 T cell immune responses *in vivo*.

The closely related proteins, CD2AP and CIN85, are scaffolding molecules that possess three tandem SH3 domains, a proline rich domain, and a C-terminal coiled-coil domain that mediates their hetero/homodimerization [8]. CD2AP was originally identified via a yeast two-hybrid screen as an interacting partner of the adhesion molecule CD2 and a dominant negative form expressed in Jurkat T cells prevented formation of the immunological synapse [9]. However, in naive T cells from AND TCR transgenic mice lacking CD2AP, formation of the immunological synapse was intact, suggesting that CD2AP functions independently of CD2 in primary T cells [10]. Interestingly, following stimulation with pMHC, activation of proximal kinases and degradation of TCR are delayed, leading to prolonged TCR activation and enhanced cytokine production *in vitro* [10]. However, the role of CD2AP in tuning of TCR signaling in T cell immune responses or the impact of altered TCR signaling quality *in vivo* caused by *Cd2ap* deficiency has not been defined due to a fatal kidney disease that develops around 3 weeks of age in *Cd2ap*^−/−^ mice [11].

Here, we generated T cell-specific *Cd2ap*^−/−^ deficient mice and show that CD2AP deficiency enhances control of chronic viral infection by augmenting antiviral T_FH_ and GC responses. In contrast, T_FH_ and GC responses were minimally altered when the mice were immunized with sheep red blood cells (SRBCs), which are prone to elicit type 2 responses [12]. TCR signaling in *Cd2ap*^−/−^ CD4 T cells was prolonged specifically under T_H_1 conditions *in vitro*, resulting in increased production of IFN-γ, while CD2AP was dispensable for temporal tuning of TCR signals in T_H_2 or T_H_17 cells. These results demonstrate that CD2AP-dependent tuning of TCR signaling in CD4 T cells is T_H_ subset-specific and that CD2AP may be an effective target to accelerate the development of protective antibodies in antiviral immune responses.

## Results

### T_FH_ differentiation is enhanced in CD2AP-deficient mice late in acute viral infection

To conduct analyses of function of CD2AP in T cells *in vivo*, we generated the *Cd2ap*-flox allele, in which expression of cre recombinase results in deletion of exon 2 (S1A Fig). This allele was bred to *Cd4*-cre to inactivate *Cd2ap* in T cells. Numbers of mature CD4 and CD8 single positive thymocytes and CD4 and CD8 T cells in the spleen were comparable between *Cd4*-cre^+^ *Cd2ap*^F/F^ and control *Cd2ap*^F/F^ mice (S1B and S1C Fig).

**Fig 1:**
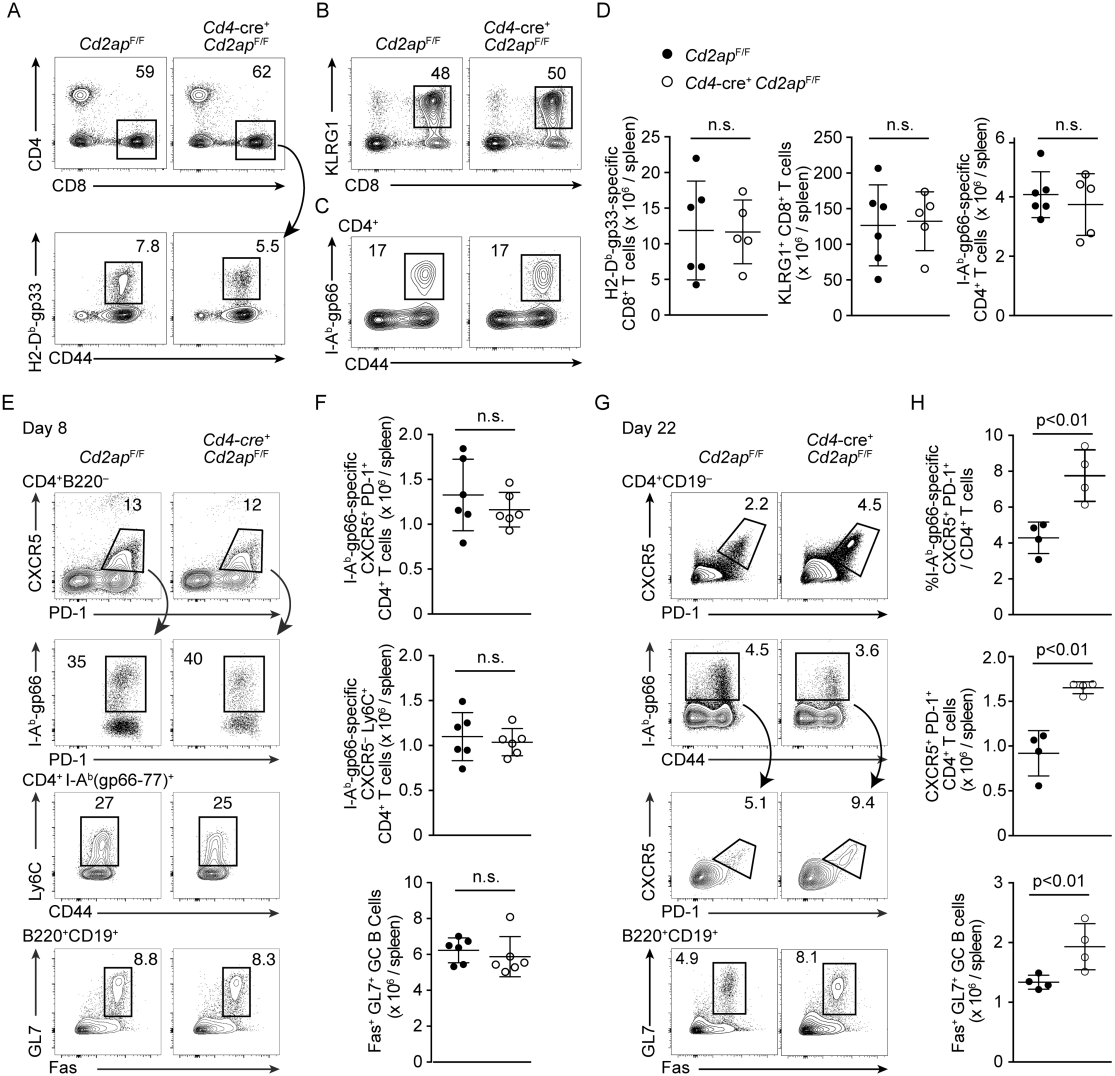
Enhanced T_FH_ and GC B cell responses in T cell-specific *Cd2ap*-deficient mice in response to acute viral infection. (A) Flow cytometric analysis of expression of CD4, CD8 and CD44 and binding of H-2D^b^(gp33-41) tetramer in splenocytes 8 days after LCMV-Armstrong infection. Total CD8 T cells and LCMV-gp33-specific CD8 T cells are shown with rectangular gates. (B) Expression of CD8 and KLRG1 in splenocytes 8 days after LCMV-Armstrong infection. (C) Expression of CD44 and binding of I-A^b^(gp66-77) tetramer in CD4^+^ splenic T cells 8 days after LCMV-Armstrong infection. LCMV-gp66-specific CD4 T cells are shown with rectangular gates. (E, G) Expression of PD-1, CXCR5 and Ly6C and binding of I-A^b^ (gp66-77)^+^ tetramer of CD4^+^ T cells and Fas and GL7 expression in B cells in the spleens of *Cd4*-cre^+^ *Cd2ap*^F/F^ and control *Cd2ap*^F/F^ mice 8 (E) and 22 (G) days after infection. T_FH_ cells and GC B cells are shown with rectangular gates. Representative plots are shown with percentages of gated cells. Statistical analyses from 4–6 mice in 2 independent experiments are shown with means and standard deviation in (D, F, H).

To define the role of CD2AP in T cells *in vivo*, we infected *Cd4*-cre^+^ *Cd2ap*^F/F^ and control *Cd2ap*^F/F^ littermate control mice with the Armstrong strain of lymphocytic choriomeningitis virus (LCMV), which elicits acute antiviral responses and is cleared by CD8 T cells. At the peak of the T cell response on day 8 after infection, no difference was seen in numbers of total CD8 T cells, KLRG1^+^ short-lived effector CD8 T cells or that of LCMV glycoprotein (gp)-specific CD8 T cells between *Cd4*-cre^+^ *Cd2ap*^F/F^ and *Cd2ap*^F/F^ mice (Fig 1A, 1B and 1D), suggesting that CD2AP is not required for normal CD8 T cell responses to acute LCMV infection. Comparable numbers of LCMV-gp-specific CD4 T cells also were detected in both genotypes eight days after infection (Fig 1C and 1D), suggesting that CD2AP is dispensable for priming and initial expansion of LCMV-specific CD4 T cells. Both *Cd2ap*^−/−^ and control LCMV-specific CD4 T cells differentiated into Ly6C^+^ T_H_1 effector cells or T_FH_ cells as defined by CXCR5 and PD-1 expression on day 8 after infection (Fig 1E and 1F). Accordingly, we did not observe a difference in either frequency or absolute number of Fas^+^ GL7^+^ GC B cells at this time point (Fig 1E and 1F). At a later time-point (day 22), however, the frequency and absolute number of total and LCMV-specific CXCR5^+^ PD-1^+^ T_FH_ cells as well as GL7^+^ Fas^+^ GC B cells was significantly increased in *Cd4*-cre^+^ *Cd2ap*^F/F^ mice (Fig 1G and 1H). These results indicate that *Cd2ap*-deficiency enhances CD4 T cell differentiation into the T_FH_ subset following initial bifurcation between T_H_1 effector and T_FH_ fates early in infection, and augments GC B cell responses.

**Fig 2:**
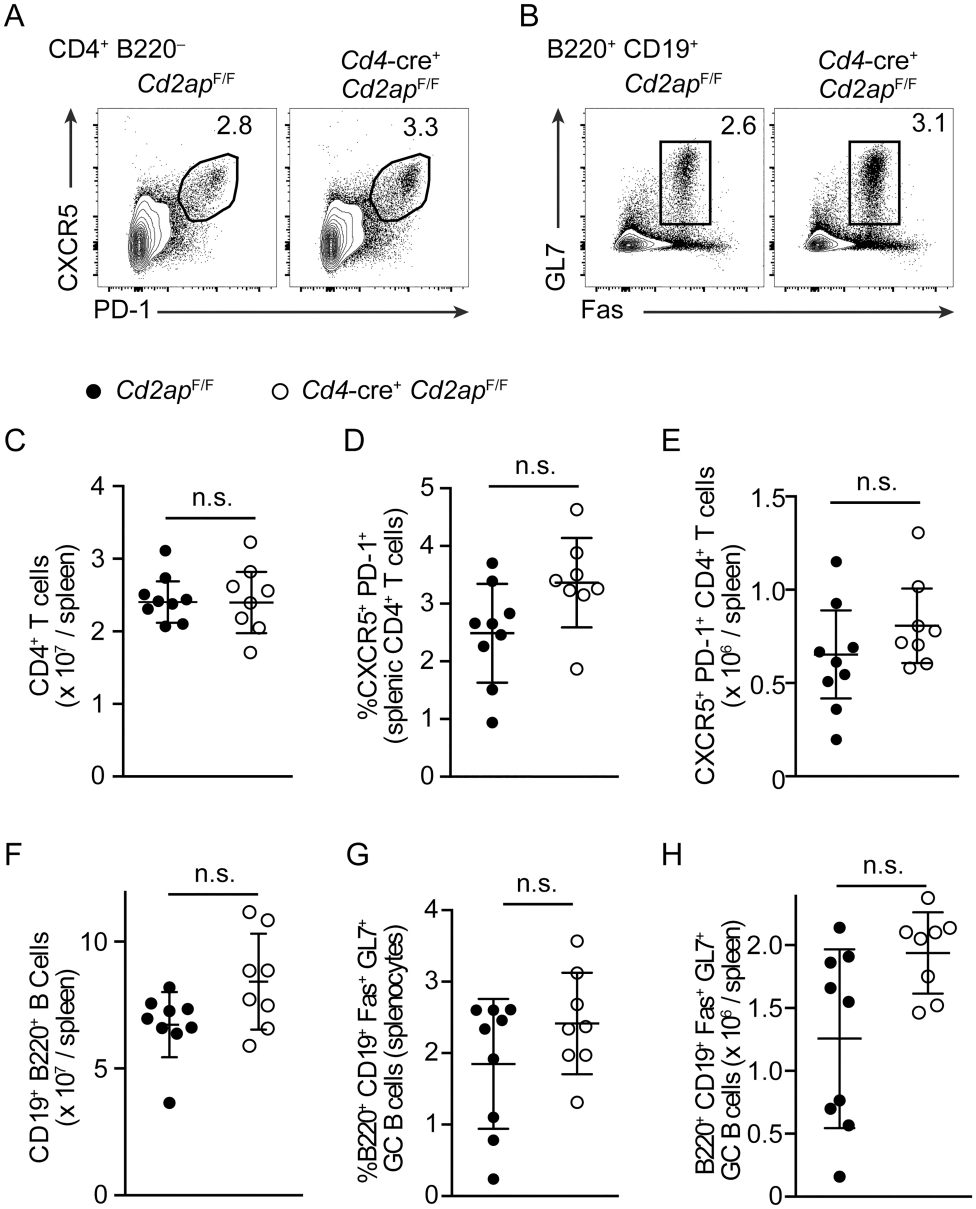
*Cd2ap* deficiency has a minimal impact on T_FH_ differentiation and GC B cell responses following immunization with SRBCs. (A, B) Flow cytometric analysis of expression of PD-1 and CXCR5 on pre-gated CD4^+^ B220^−^ T cells (A) and GL7 and Fas expression on CD19^+^ B220^+^ B Cells (B) 12 days following SRBC immunization. (C-E) Numbers and frequencies of total CD4 T cells and CXCR5^+^ PD-1^+^ T_FH_ in the spleen of *Cd4*-cre^+^ *Cd2ap*^F/F^ and control *Cd2ap*^F/F^ mice 12 days after immunization with SRBCs. (F-H) Numbers and frequencies of total B cells and Fas^+^ GL7^+^ GC B cells in the spleen of *Cd4*-cre^+^ *Cd2ap*^F/F^ and control *Cd2ap*^F/F^ mice 12 days after immunization with SRBCs. Data are pooled from 2 independent experiments shown as means and standard deviation.

To determine whether T_FH_ differentiation of *Cd2ap*^−/−^ CD4 T cells is also enhanced by immunization that induces other types of CD4 T cell responses, we analyzed the GC response after immunization of *Cd4*-cre^+^ *Cd2ap*^F/F^ and *Cd2ap*^F/F^ mice with SRBCs, which predominantly induces development of IL-4 producing CD4 T cells [13, 14]. While in the absence of CD2AP, a statistically insignificant trend in increased T_FH_ and GC responses was seen at day 12 following immunization, a relatively late time point when antigen levels are declining [15] (Fig 2A–2H), numbers of T_FH_ and GC B cells were comparable between *Cd4*-cre^+^ *Cd2ap*^F/F^ and *Cd2ap*^F/F^ mice both at a relatively early time point, day 6, and a later time point day 22 (S2A–S2D Fig). In addition, we found no significant differences in T_FH_ and GC B cell numbers between *Cd4*-cre^+^ *Cd2ap*^F/F^ and *Cd2ap*^F/F^ mice following immunization with NP-CGG precipitated in aluminum salts (S2E and S2F Fig), which also induces a preferential T_H_2 biased CD4 T cell response [16, 17]. These results suggested that CD2AP suppresses CD4 T cell differentiation towards the T_FH_ subset dependent on the context in which the response is induced.

### Enhanced control of chronic LCMV infection in T cell-specific *Cd2ap*-deficient mice

**Fig 3:**
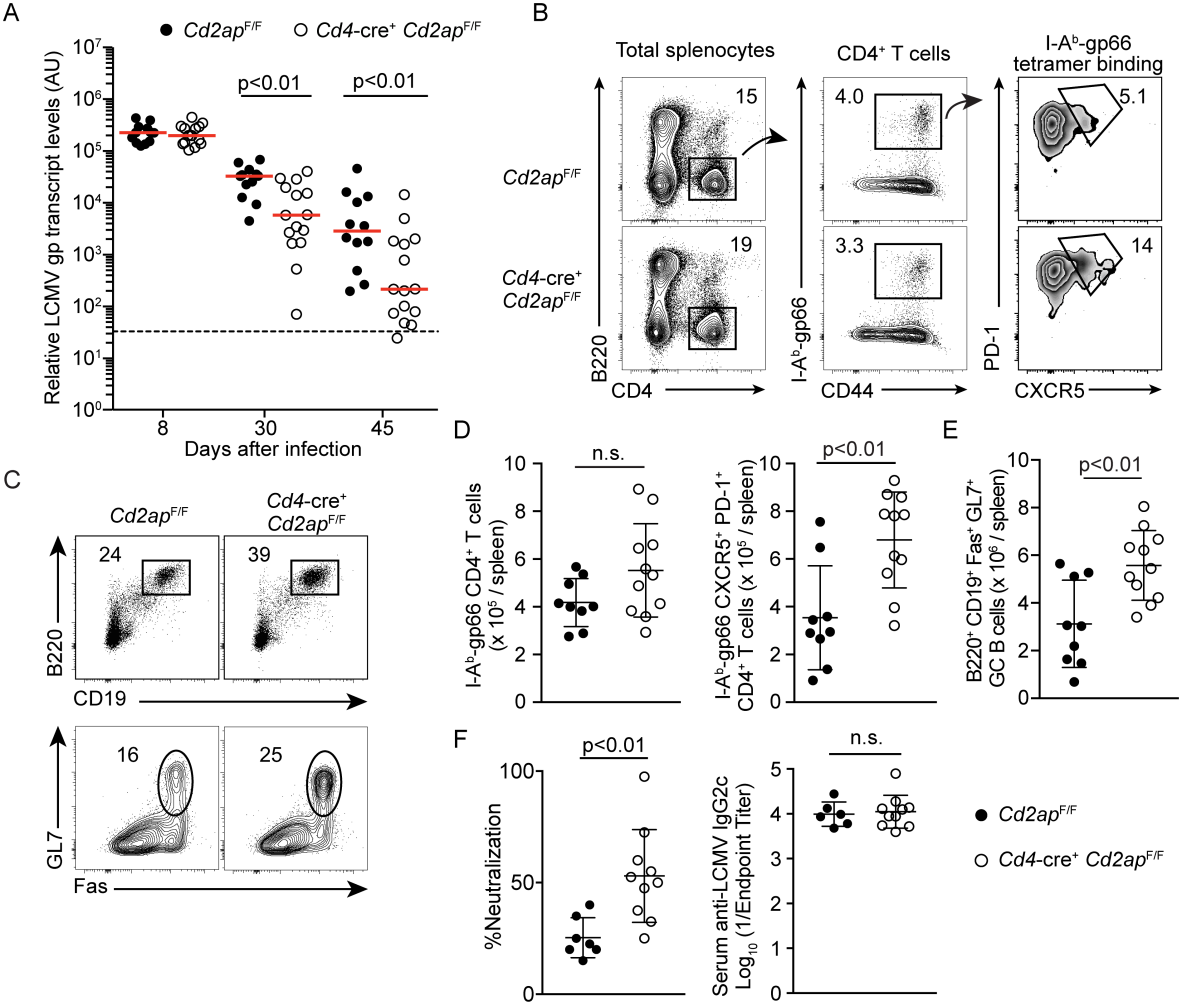
Enhanced control of LCMV-c13 infection in *Cd4*-cre^+^ *Cd2ap*^F/F^ is associated with elevated T_FH_ response. (A) Analysis of LCMV abundance in plasma in mice as determined by LCMV *gp* transcript levels. Horizontal bars indicate medians. The limit of detection is shown by a dashed line. Statistical significance was tested by Mann Whitney U-test. (B-E) Expression of B220, GL7, Fas, CD4, CD44, PD-1 and CXCR5 and binding of I-A^b^ (gp66-77) tetramer of splenocytes from *Cd2ap*^F/F^ and *Cd4*-cre^+^ *Cd2ap*^F/F^ mice 22 days after LCMV-c13 infection. Representative plots are shown with percentages of gated cells. Statistical analyses from at least 7–10 mice per genotype in 2 independent experiments are shown with means and standard deviation in (D) and (E). (F) (Left) Neutralizing activity and (Right) anti-LCMV IgG2c antibody titers of plasma from *Cd2ap*^F/F^ and *Cd4*-cre^+^ *Cd2ap*^F/F^ mice 60 days after LCMV-c13 infection. Reduction in focus forming units (FFU) in the presence of 1:10 dilution of plasma compared to untreated control (%Neutralization) is shown with mean and standard deviation. *Cd2ap*^F/F^: n = 7, *Cd4*-cre^+^ *Cd2ap*^F/F^: n = 9.

While we observed enhanced T_FH_ differentiation in *Cd2ap*-deficient mice to LCMV-Armstrong, CD8 CTLs rather than LCMV-specific antibodies are required for control of acute LCMV infection [18]. To determine whether enhanced T_FH_ differentiation seen in CD2AP deficiency had an impact on antiviral antibody-mediated immunity, we utilized the LCMV-clone13 (LCMV-c13) model of chronic viral infection since T_FH_-dependent high affinity antibody responses in the GCs and class-switch recombination to IgG2a/2c subclasses are required for control of the infection [19–21]. After infection, LCMV replicated and established viremia to a similar extent in *Cd4*-cre^+^ *Cd2ap*^F/F^ and control cre^−^*Cd2ap*^F/F^ mice on day 8 (Fig 3A). In control cre^−^*Cd2ap*^F/F^ mice, viral abundance, as determined by the quantities of the LCMV *gp* transcript in the plasma of mice, started to gradually decline around day 30 (Fig 3A), coinciding with expansion of T_FH_ and GC B cells in response to a surge of IL-6 production by follicular dendritic cells [19]. The decline in LCMV abundance was significantly accelerated in *Cd4*-cre^+^ *Cd2ap*^F/F^ mice, with the viral titers below the limit of detection in some *Cd4*-cre^+^ *Cd2ap*^F/F^ mice by day 45 (Fig 3A and S3A Fig). Consistent with the accelerated clearance of LCMV, the frequencies and numbers of total and LCMV gp-specific T_FH_ cells in the spleen 22 days after infection were significantly increased (Fig 3B and 3D; S3B and S3C Fig), although total numbers of LCMV-specific CD4 T cells were not changed in *Cd4*-cre^+^ *Cd2ap*^F/F^ mice compared to control mice (Fig 3D). Accordingly, frequencies and numbers of GC B cells were significantly increased in *Cd4*-cre^+^ *Cd2ap*^F/F^ mice compared to control mice (Fig 3C and 3E; S3D Fig). It has been demonstrated that T follicular regulatory (T_FR_) cells can suppress the GC response [22]; thus we analyzed the frequency of Foxp3^+^ cells within the T_FH_ compartment, however, we did not find differences between genotypes indicating T_FR_ differentiation was unimpaired (S3E and S3F Fig). Moreover, plasma from *Cd4*-cre^+^ *Cd2ap*^F/F^ mice exhibited elevated neutralizing activity against LCMV *in vitro*, while titers of total anti-LCMV antibodies with dominant IgG2c and sub-dominant IgG1 subclasses were comparable between *Cd4*-cre^+^ *Cd2ap*^F/F^ and control *Cd2ap*^F/F^ mice (Fig 3F; S3G Fig). As a measure of GC output we also analyzed the number of memory B cells in the spleen and plasma cells in the bone marrow at 60 days following LCMV-c13 infection and observed no difference in overall numbers between *Cd4*-cre^+^ *Cd2ap*^F/F^ and control *Cd2ap*^F/F^, suggesting enhanced acquisition of mutations rather than total output contributed to the phenotype (S4 Fig). Given the importance of CD4 T cell help in promoting long-lived polyfunctional CD8 T cells we also analyzed the CD8 T cell response at day 22 [23, 24]. Importantly, we did not detect differences in overall CD8 T cell numbers or LCMV-specific H-2D^b^(gp33-41) tetramer binding T cells in *Cd4*-cre^+^ *Cd2ap*^F/F^ mice compared to control mice (S5A and S5B Fig). In addition, we did not observe differences in the polyfunctionality of LCMV-specific CD8 T cells as determined by IFN-γ and TNF-α production (S5C Fig). These results indicate that while inactivation of CD2AP only minimally affects the CD8 T cell response, it enhances T_FH_ differentiation and generation of neutralizing antiviral antibodies by augmenting B cell help.

### CD2AP deficiency promotes T_FH_ differentiation in a cell-intrinsic manner

**Fig 4:**
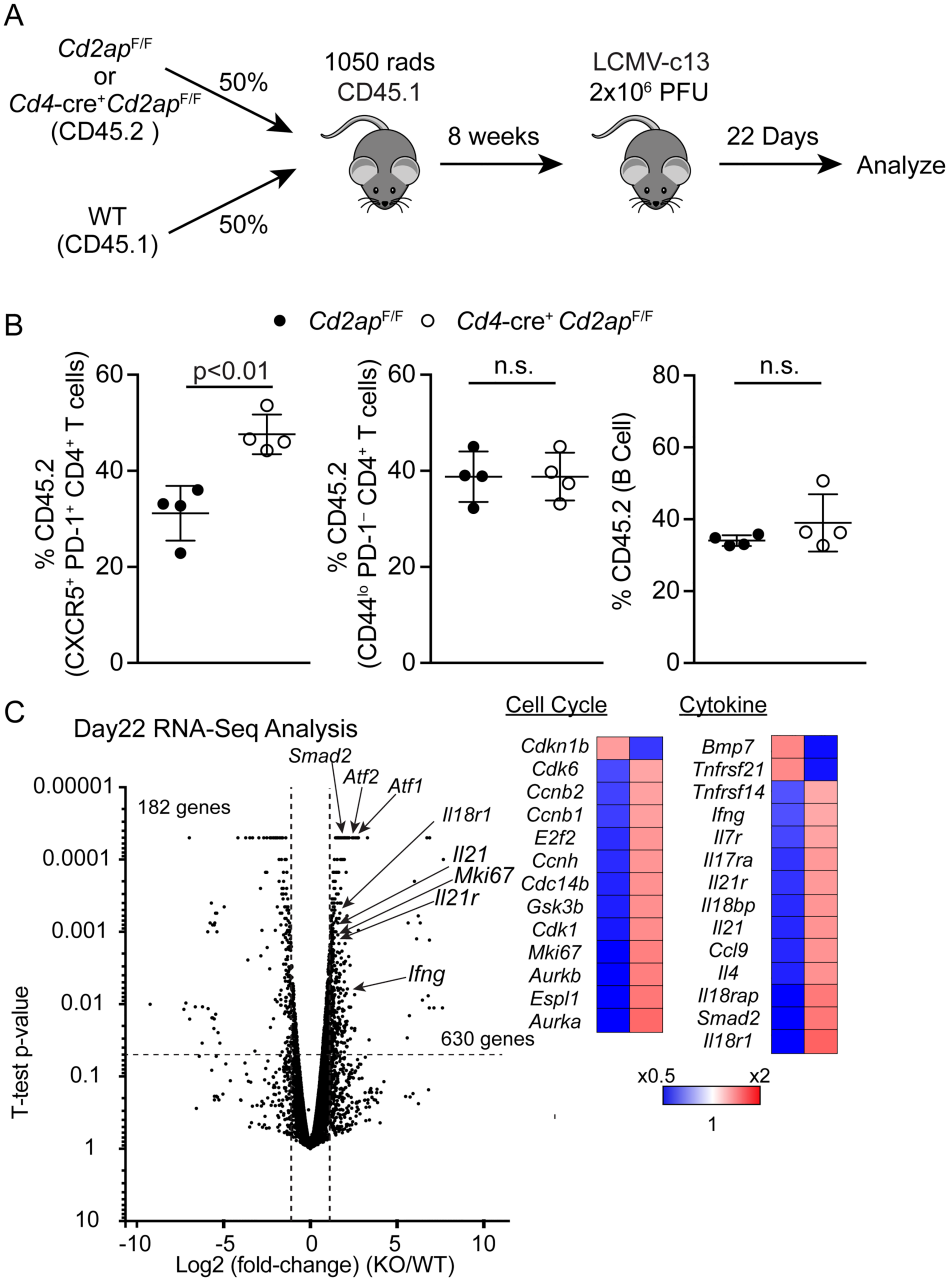
*Cd2ap*-deficiency enhances T_FH_ differentiation in a cell-intrinsic manner. (A) Schematic of experimental strategy for the generation of mixed BM chimeras and subsequent analysis of anti-LCMV responses. (B) Contribution of CD45.2 *Cd2ap*^F/F^ or *Cd4*-cre^+^ *Cd2ap*^F/F^ cells to B cells, activated CD4 T cells and T_FH_ cells in the mixed BM chimeras 22 days after LCMV-c13 infection. Representative Data from 2 independent experiments (n = 4 of each genotype) are shown with means and standard deviations. Statistical difference was tested by Student’s *t*-test. (C) A volcano plot showing differentially expressed genes between Cd2ap-deficient (KO, n = 2) and -sufficient (WT, n = 2) T_FH_ cells harvested from mixed BM chimeras 22 days after infection. Genes that were differentially expressed by >2-fold are shown.

To determine whether the increased T_FH_ differentiation in *Cd4*-cre^+^ *Cd2ap*^F/F^ mice was cell-intrinsic, we generated BM chimeras by transferring a mixture of BM cells from *Cd4*-cre^+^ *Cd2ap*^F/F^ or cre^−^*Cd2ap*^F/F^ mice (CD45.2) and CD45.1 WT mice into lethally irradiated CD45.1 WT mice. Following reconstitution of donor-derived hematopoiesis, the recipient mice were infected with LCMV-c13 and contribution of *Cd2ap*-deficient and -sufficient cells to the T_FH_ compartment was examined 22 days after infection (Fig 4A). While the percentages of CD45.2 cells in the B cell and CD44^lo^ PD-1^−^ naive CD4 T cell compartments were equivalent between chimeras reconstituted with *Cd4*-cre^+^ *Cd2ap*^F/F^ or cre^−^*Cd2ap*^F/F^ BM cells, the contribution of CD45.2 cells to the CXCR5^+^ PD-1^+^ T_FH_ pool was significantly greater in chimeras receiving *Cd4*-cre^+^ *Cd2ap*^F/F^ compared to control chimeras receiving cre^−^*Cd2ap*^F/F^ BM cells (Fig 4B). In addition to the quantitative increase in T_FH_ numbers in *Cd4*-cre^+^ *Cd2ap*^F/F^ mice, *Cd2ap*-deficient T_FH_ cells expressed elevated levels of genes that are associated with T_FH_ functions, including *Il21*, *Il21r* and *Il17ra*, and also those associating with active cell cycling (Fig 4C) [25]. We confirmed these results by analysis of frequencies of Ki67^+^ cells within T_FH_ cells and found them to be increased in KO cells compared to WT (S3H Fig). In contrast to these changes, we did not observe changes in expression of T_FH_ associated molecules such as ICOS and OX-40, suggesting these costimulatory pathways might not contribute to the observed phenotype (S3I Fig). These results suggest that *Cd2ap*-deficient CD4 T cells are maintaining highly activated states, potentially through enhanced TCR-dependent signaling, and establish a stable T_FH_ program potentially by the IL-21-IL-21R feed-forward loop and thus better support GC B cell responses against viral infection.

### CD2AP-deficiency causes sustained TCR activation specifically in T_H_1 cells

**Fig 5:**
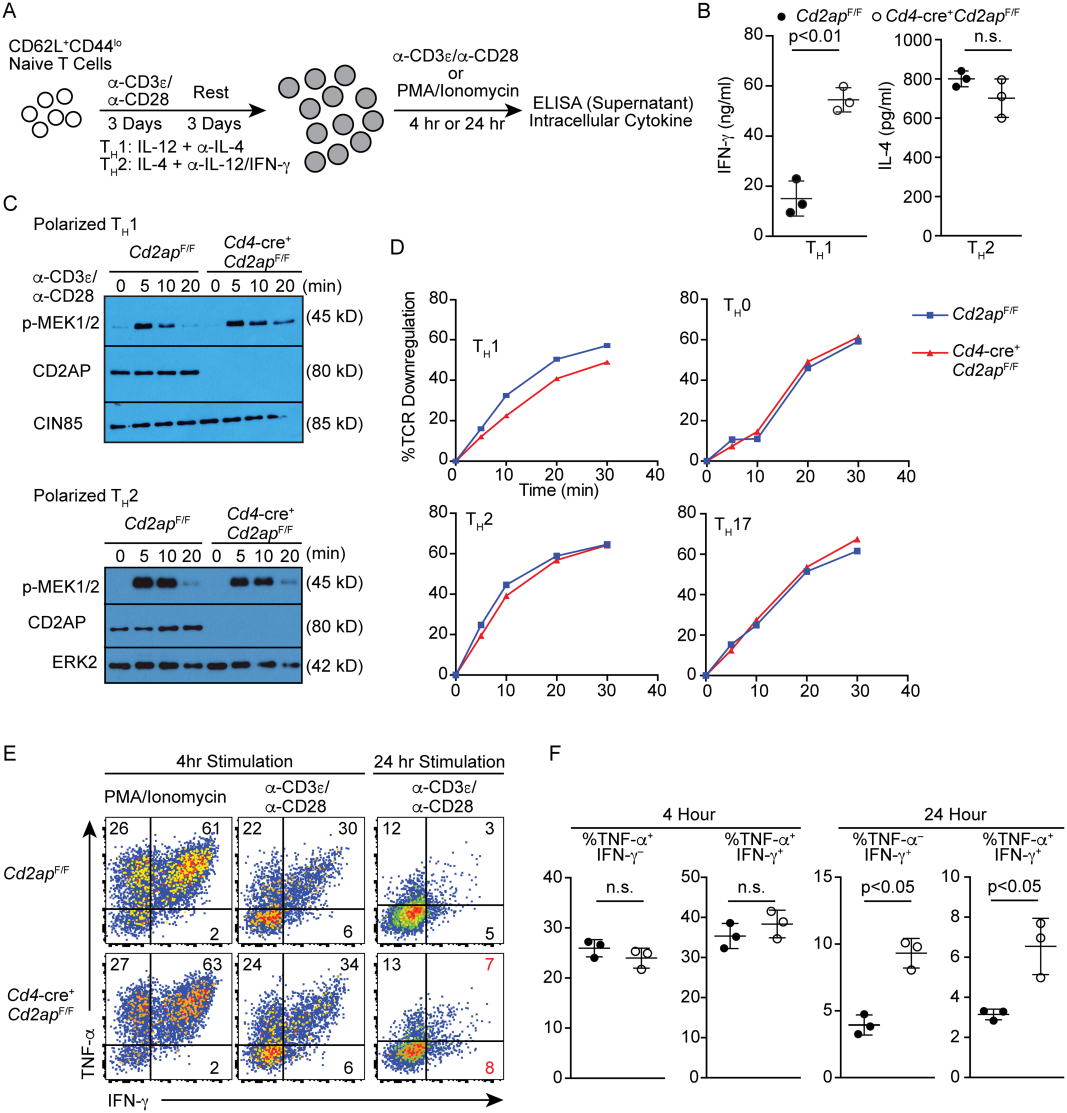
*Cd2ap*-deficiency causes sustained TCR signaling specifically in T_H_1 cells *in vitro*. (A) Schematic of experimental strategy for culture of CD4 T cells. (B) Concentration of IFN-γ or IL-4 in supernatant measured by ELISA 24 hours after re-stimulation of polarized *Cd2ap*^F/F^ and *Cd4*-cre^+^ *Cd2ap*^F/F^ T_H_1 cells and T_H_2 cells with plate-bound anti-CD3/anti-CD28 antibodies. (C) Immunoblotting showing phosphorylation of MEK1/2 in polarized *Cd2ap*^F/F^ and *Cd4*-cre^+^ *Cd2ap*^F/F^ T_H_1 or T_H_2 cells following re-stimulation with plate-bound anti-CD3/anti-CD28 for the indicated times at 37°C. Anti-CD2AP, -CIN85 and ERK were used as control. Data are representative of three experiments. (D) Downregulation of surface TCR of *Cd2ap*^F/F^ and *Cd4*-cre^+^ *Cd2ap*^F/F^ T cells that were polarized under indicated conditions. Cells were stimulated with plate-bound anti-CD3 and surface TCR levels were quantitated at the indicated time points. Data are representative of three experiments. (E, F) Intracellular staining for IFN-γ and TNF-α of polarized *Cd2ap*^F/F^ and *Cd4*-cre^+^ *Cd2ap*^F/F^ T_H_1 cells after re-stimulation with PMA and Ionomycin or plate-bound anti-CD3 and anti-CD28 for 4 or 24 hours in the presence of Brefeldin A for the last 2 hours before harvest. Representative plots (E) and statistical analyses with means and standard deviations (F) from three experiments are shown.

Our data demonstrated that T_FH_ differentiation was enhanced by *Cd2ap*-deficiency specifically in viral infection, but not in immunization with SRBCs or NP-CGG in alum. These results suggest that CD2AP regulates TCR signaling in a T_H_-subset-specific manner. To dissect the subset-specific role of CD2AP in CD4 T cells, we activated naive CD4 T cells from *Cd4*-cre^+^ *Cd2ap*^F/F^ and control *Cd2ap*^F/F^ cells under polarizing conditions that promote generation of T_H_1 or T_H_2 cells and compared their responses to antigen receptor stimulation (Fig 5A). While production of IL-4 under T_H_2 conditions was comparable between *Cd2ap*-deficient and -sufficient CD4 T cells, the production of IFN-γ was increased by 3-fold in *Cd2ap*-deficient T_H_1 cells compared to control cells (Fig 5B). Consistently, activation of the MAPK pathway was prolonged specifically in *Cd2ap*-deficient T_H_1 compared to control T_H_1 cells, whereas we did not observe the difference in T_H_2 cells (Fig 5C). Previous studies of CD4 T cells using germline *Cd2ap*-deficient mice showed that *Cd2ap*^−/−^ TCR-transgenic naive T cells are defective in TCR downregulation [10]. In polarized T_H_1 cells, downmodulation of surface TCRs was significantly impaired in the absence of CD2AP, while the difference was not observed under T_H_0, T_H_2 or T_H_17 conditions between *Cd2ap*-deficient and -sufficient CD4 T cells (Fig 5D). Furthermore, when *Cd2ap*-deficient and control T_H_1 cells were re-stimulated with plate-bound anti-CD3 and anti-CD28 antibodies or with PMA and Ionomycin for 4 hours, similar frequencies of cells expressed IFN-γ and TNF-α (Fig 5E and 5F). However, when they were stimulated with plate-bound anti-CD3 and anti-CD28 antibodies for 24 hours, the frequencies of cells that expressed IFN-γ or TNF-α during the last 2 hours of the stimulation were significantly increased in *Cd2ap*-deficient T_H_1 cells relative to WT T_H_1 cells presumably due to prolonged retention of TCR on the cell surface, which was crosslinked by the plate-bound antibodies (Fig 5E and 5F). These results indicate that CD2AP deficiency prolongs TCR signaling specifically in CD4 T cells under T_H_1 conditions.

### CIN85 plays a non-redundant role in T cell-dependent control of LCMV infection

**Fig 6:**
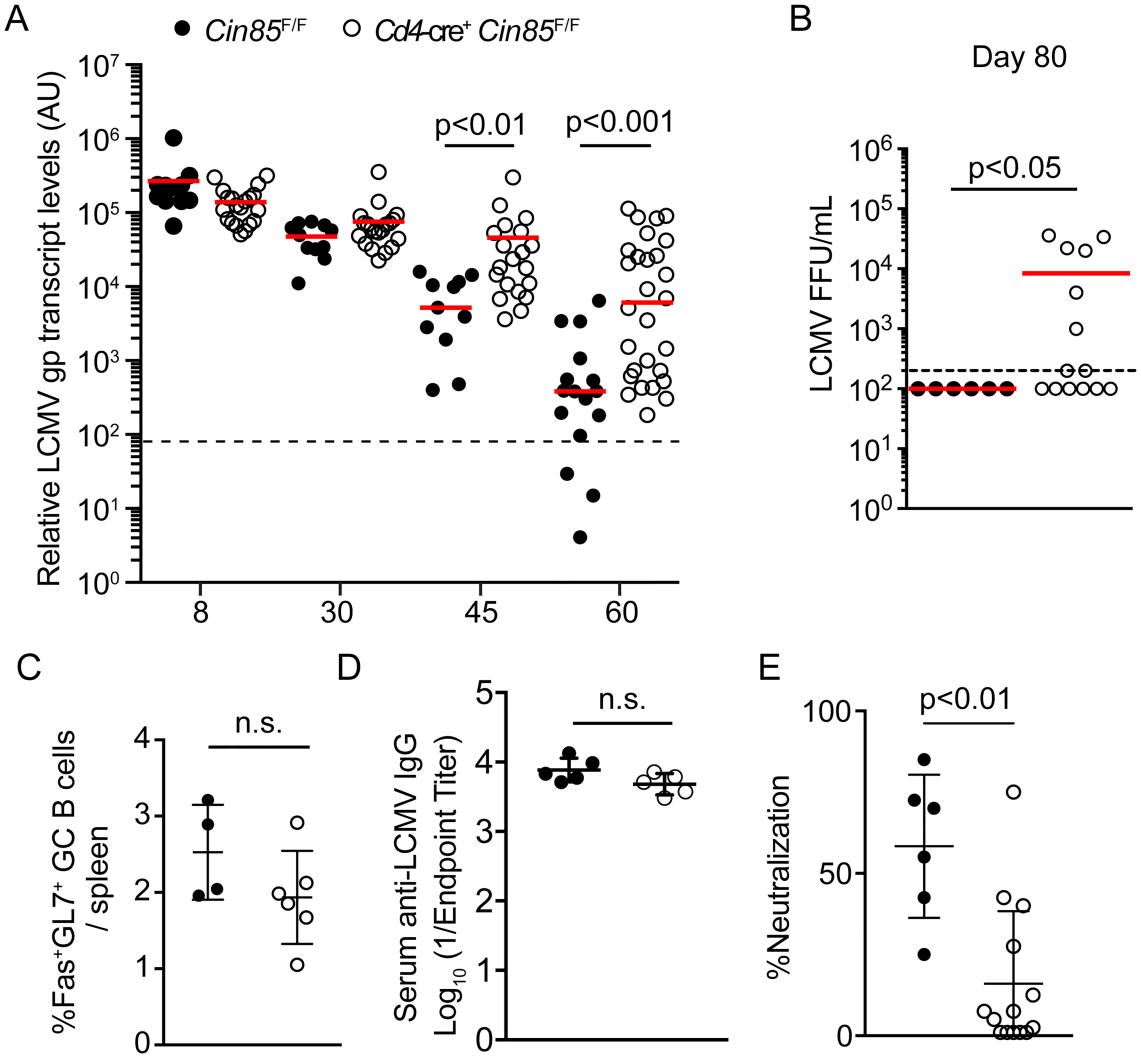
*Cin85*-Deficiency results in defective clearance of LCMV-c13 and is correlated with decreased neutralizing antibody titer. (A, B) Analysis of LCMV abundance in plasma in *Cin85*^F/F^ and *Cd4*-cre^+^ *Cin85*^F/F^ mice as determined by LCMV *gp* transcript levels (A) or focus forming assay (B) at day 80. Horizontal lines indicate median. The limit of detection is shown by dashed lines. Statistical significance was tested by Mann Whitney U-test. (C) Frequencies of Fas^+^ GL7^+^ B220^+^ GC B cells at day 35 after LCMV-c13 infection. (D) anti-LCMV IgG antibody titers of plasma from *Cin85*^F/F^ and *Cd4*-cre^+^ *Cin85*^F/F^ mice 30 days after LCMV-c13 infection. (E) Neutralizing activity of plasma from *Cd2ap*^F/F^ and *Cd4*-cre^+^ *Cd2ap*^F/F^ mice 80 days after LCMV-c13 infection. Reduction in focus forming units (FFU) in the presence of 1:5 dilution of plasma compared to untreated control (% Neutralization) is shown with mean and standard deviation. Student *t*-test. Data combined from 3 independent experiments are shown with n = 3–4 mice per genotype.

CIN85 and CD2AP are closely related and may have redundant functions [8,12]. To test this, we also generated mice in which T cells are deficient for CIN85 by breeding a *Cin85*-flox allele to both *Cd4*-cre [26]. *Cd4*-cre^+^ *Cin85*^F/F^ mice exhibited no differences in CD4 T and CD8 T cell response to acute LCMV infection (S6 Fig). However, in contrast to *Cd4*-cre^−^*Cd2ap*^F/F^ mice, *Cd4*-cre^+^ *Cin85*^F/F^ mice were unable to control chronic LCMV infection with significantly higher viral burden in the peripheral blood at days 45 and 60 after infection compared to cre^−^ littermate controls (Fig 6A). In addition, while all WT mice had cleared virus from the plasma by day 80, approximately half of *Cd4*-cre^+^ *Cin85*^F/F^ still exhibited high viral load (Fig 6B). The clearance phenotype was more pronounced when we infected *Vav1*-icre^+^ *Cin85*^F/F^ mice, in which all hematopoietic cells, including B cells, were deficient for *Cin85* (S7A Fig), suggesting CIN85 plays additional roles in other hematopoietic cells in the context of LCMV-c13 infection, potentially through B cells as previously reported [26]. However, when we analyzed mice at day 30 following infection we did not find any significant differences in either frequency or absolute number of CD8 T cell, T_FH_, or GC response (S7B–S7D Fig). Consistently, when we analyzed *Cd4*-cre^+^ *Cin85*^F/F^ mice, the frequency of Fas^+^ GL7^+^ GC B cells was only marginally, but insignificantly, decreased in *Cd4*-cre^+^ *Cin85*^F/F^ mice compared to control *Cin85*^F/F^ mice (Fig 6C) with anti-LCMV IgG titers unchanged, suggesting no severe defects in the GC response (Fig 6D). However, plasma from infected *Cd4*-cre^+^ *Cin85*^F/F^ mice exhibited a significantly reduced neutralizing activity compared to plasma from cre^−^ littermate controls (Fig 6E). These results indicate that CIN85 is required for protective antibody responses in a T cell-dependent manner presumably through controlling T_FH_ function and potentially the quality of help provided to GC B cells with respect to generation of high-affinity neutralizing antibodies required for clearance of chronic LCMV [27]. These results suggest that although CD2AP and CIN85 share significant homology, their functions in antiviral CD4 T cell responses are non-redundant, and potentially antagonistic.

## Discussion

This study demonstrated that deletion of CD2AP in T cells results in skewing of CD4 T cell differentiation towards T_FH_ cells in response to viral infection, leading to enhanced control of LCMV that requires GC-derived high affinity antibody responses [19, 21, 28]. T_FH_ differentiation was correlated with sustained TCR signaling under T_H_1 conditions, while TCR signaling *in vitro* under non-T_H_1 conditions was not altered. Thus, our work revealed a specific role of CD2AP in subset-specific CD4 T cell responses.

Sustained TCR stimulation during chronic LCMV infection or in the cancer microenvironment causes deregulation of CD8 T cells, a phenomenon known as exhaustion [1], [29]. Frequent interactions with cognate pMHC-I result in the persistent upregulation of several inhibitory receptors which act to dampen T cell proliferation and effector functions, a hallmark of the “exhausted state” [2, 29]. However, the impact of sustained TCR stimulation on the function of CD4 T cells has been less clearly understood. In chronic LCMV infection, CD4 T cells exhibit less IL-2 production and increased IL-10 production, a phenomenon that is similar in nature to CD8 T cell “exhaustion” [3,30–32]. However, these CD4 T cells with the altered activation state acquire the capability of producing IL-21, a key cytokine that enhances the GC response and also supports the CD8 T cell response; both are required for control of the viral infection [4, 31–33]. Thus, although sustained TCR signaling compromises CD8 T cell functions, CD4 T cells are able to tolerate sustained signaling through TCR to mediate pathogen control.

Several recent studies indicate that during chronic LCMV infection, CD4 T cells exhibit a relatively unique propensity to acquire T_FH_ features, a process that is dependent on continuous antigen stimulation [5, 34]. The acquisition of T_FH_ phenotype in chronic infection appears to be different compared to acute LCMV infection [35]. Interestingly, in late phases >day 20 of LCMV-c13 infection B cells do not appear to be absolutely required for the development of CXCR5^+^ cells, suggesting other types of antigen presenting cells could contribute to the sustained T_FH_ response as this does not occur in MHCII KO [34]. Preferential T_FH_ accumulation has also been shown to be dependent on type-I Interferon [6, 36] which has not been explicitly observed in acute contexts, via a cell-extrinsic mechanism, suggesting other soluble or cell-associated factors could have a more direct influence [36]. These results illustrate the complexity in direction of the CD4 T cell response, and illustrate the variety of mechanisms that influence the humoral response in response to the nature of the insulting pathogen. However, in several contexts it appears that modulation of TCR affinity and or signal duration impacts the differentiation of T_FH_ cells, supporting a more broadly applicable role for the TCR.

Our study with CD2AP-deficient T cells confirm and extend previous studies that suggest that sustained TCR signaling promotes T_FH_ differentiation [37, 38]. Specifically, it was shown following infection by *Listeria monocytogenes* that TCR:pMHCII dwell time correlated well with GC-T_FH_ differentiation [37]. In addition, in antigen-specific cells elicited by immunization with pigeon cytochrome c, T_FH_ phenotype cells tended to have higher affinity for tetramer compared to non-T_FH_ cells, and transfer of TCR transgenic cells with higher affinity for pMHCII revealed a preference for T_FH_ differentiation [38]. One potential explanation is that T_FH_ and T_H_1 cells may share a transitional stage, at which sustained TCR signaling may restrict T-bet upregulation to direct the activated CD4 T cells into the T_FH_ subset, which favors low-T-bet [39], while T_FH_ differentiation during a type-2 immune response may require a distinct mechanism. This may explain why T_FH_ differentiation is enhanced when CD4 T cells receive sustained TCR stimulation as antigen levels decline. Interestingly, enhanced T_FH_ differentiation and increased IL-21 expression by *Cd2ap*-deficient CD4 T cells did not noticeably alter the CD8 T cell response, which is also dependent on CD4 T cell help. These results potentially indicate that the nature of CD4 T cell help to B cells and CD8 T cells may be independent.

While downregulation of the TCR and subsequent termination of TCR downstream signaling is broadly shared by T cells, our results indicate that CD2AP-dependent regulation of TCR signaling duration is T_H_1-specific *in vitro*, which correlates with enhanced T_FH_ differentiation in LCMV infection. However, it still remains unclear whether the *in vivo* functions of CD2AP are completely explained by TCR signaling via the immunological synapse, interactions with CD2, or potentially via signaling via other cytokines such as IL-21 or Type I interferon and is an area of further investigation. Notably, a recent study reported T_H_2-specific regulation of the duration of TCR signaling by the Rab-GTPase Dennd1b [40]. Dennd1b-deficiency in CD4 T cells results in sustained TCR signaling and increased cytokine production specifically under T_H_2 conditions. Furthermore, a *DENND1B* SNP in human, causing reduced expression of its protein, is associated with asthma. These two studies, contrasting lineage-specific requirement for CD2AP and Dennd1b in controlling TCR signaling in T_H_1 and T_H_2 cells, respectively, imply that the control of TCR signaling may be uniquely regulated by different CD4 effector subsets. Despite strong correlations between sustained duration of TCR signaling *in vitro* and enhanced T cell polarization *in vivo* in Cd2ap-deficient mice and Debbd1b-deficient mice, however, there may be additional mechanisms, such as cell-cell contact or signaling through CD2, by which T cell polarization are enhanced in these genetically modified mice.

Our study highlights CD2AP as a potential target for immunotherapy to enhance B cell immune responses. Given the importance of high-affinity neutralizing antibodies in the clearance of both acute and chronic viral infections or vaccines, boosting the GC response is an attractive therapeutic target. The development of small molecules that inhibit the function of CD2AP in the context of viral infection or in the context of vaccines, could enhance the antibody response. T_FH_ responses, however, have also been associated with the development of autoantibodies and autoimmunity.

Collectively, our results demonstrate that the quality of TCR signaling is regulated by distinct mechanisms in CD4 effector T cells, and regulates differentiation of antigen-specific CD4 T cells towards distinct subsets.

## Materials and methods

### Ethics statement

Overall care and use of the animals was consistent with *The guide for the Care and Use of Laboratory Animals* from the National Research Council and the USDA *Animal Care Resource Guide* and were performed according to a protocol approved by Washington University’s Animal Studies Committee under a protocol number of 20150187, which was approved on 10/26/2015 and expires 10/9/2018. Euthanasia procedures are consistent with the “AVMA guidelines for the Euthanasia of Animals 2013 edition.”

### Mice

C57BL/6N and B6-CD45.1 mice were purchased from Charles River Laboratory. *Cd4*-cre mice in the C57BL/6 background were purchased from the Jackson Laboratory. A *Cin85* (*Sh3kbp1*)-floxed allele was described previously [26]. To generate a *Cd2ap*-flox allele, a genomic fragment containing *Cd2ap* exon2 was flanked with two *loxP* sites and subcloned into a vector containing a loxP-flanked neomycin resistant gene, a diphtheria toxin A (DTA) gene, and a 6.0-kb 5’ and a 2.1-kb 3’ homology arm. The targeting vector was electroporated into C57BL/6-derived Bruce4 embryonic stem (ES) cells. After the selection with G418 (Thermofisher), correctly targeted clones were identified by PCR. The neomycin resistant gene was removed by a transient transfection of a *cre* expression vector. The targeted ES clones were injected into blastocysts of BALB/c mice. The chimeric mice were crossed with C57BL/6 mice to obtain germline transmission. To generate BM chimeras, B6-CD45.1 mice were lethally irradiated (10.5 Gy) and reconstituted with donor BM cells for at least 8 weeks before experiments. All mice were housed in a specific pathogen-free facility at Washington University in St. Louis, and were analyzed at 8 to 10 weeks of age, unless stated otherwise. Both sexes were included without randomization or blinding.

### LCMV infection and immunization

Mice were infected with 2 x 10^5^ plaque-forming units (PFU) of LCMV-Armstrong strain via the intraperitoneal route or 2 x 10^6^ (PFU) of LCMV-c13 by intravenous injection. For the quantification of plasma viral load, RNA was extracted from 10 μL of plasma using Trizol (Life Technologies). Before RNA extraction a spike-in of RNA extracted from 293T cells (American Type Culture Collection, ATCC) expressing *gfp* mRNA was added to the plasma samples. The amounts of the LCMV GP transcript relative to that of ‘‘spiked- in” *gfp* RNA were determined by real-time qRT-PCR as previously described [21, 41]. For plasma viral titers, plasma was frozen at -80°C before performing plaque assay on Vero cells as previously described. Briefly, 7.5 x 10^5^ Vero cells (ATCC) were plated per well of a 6-well plate 24 hour prior to incubation with serial 10-fold dilutions of plasma in 200 μL of MEM/1%FBS (Thermofisher) for 1 hour at 37°C. Following the incubation Vero cells were overlaid with 0.5% Agarose (Thermofisher) solution in complete MEM and incubated for 5–6 days. Plaques were visualized following fixation in 1% PFA (Electron Microscopy Sciences) and staining with 0.1% crystal violet (Sigma).

*In vitro* neutralization assay was performed according to a published protocol with some modifications [27]. Heat-inactivated (1 hr at 55°C) plasma were serially diluted with media and incubated with an equal volume of viral supernatant containing ~30–40 focus forming units (FFU) of LCMV clone 13 at 37°C for 90 min. 4 x 10^4^ MC57G cells (ATCC) were added to the virus mixture and once the cells had adhered to the plate ~4 hours, were overlaid with a 1% methylcellulose solution in completed DMEM. 40 hours following infection, overlay was removed, and cells were fixed with 4% PFA for 1 hour at RT. Cells were permeabilized with 0.5% Triton X-100 in PBS and blocked with 10% FBS in PBS. Cells were stained with 1ug/mL of anti-LCMV NP (VL-4; BioXCell) for 1 hour at RT followed by anti-rat IgG HRP. Foci were developed using KPL TrueBlue Substrate.

Mice were immunized with 8–10 x 10^8^ SRBC (Lampire) in 200 μL or with 100μg of NP-CGG (Biosearch Technologies) precipitated Potassium Aluminum Sulfate (Sigma), via the intraperitoneal route.

### Flow cytometry and cell sorting

Single-cell suspensions were prepared by manual disruption of spleens with frosted glass slides. Absolute cell counts were determined using Vi-Cell (Becton-Dickson). The following monoclonal antibodies were used: anti-CD4 (GK1.5; Biolegend), anti-CD8a (53–6.7; Biolegend), anti-CD25 (PC61; Biolegend), anti-CD44 (IM7; Biolegend), anti-CD45.2 (104; Biolegend), anti-CD45R (B220) (RA3-6B2; Biolegend), anti-CD62L (MEL-14; Biolegend), anti-CD95 (Fas) (Jo2; BD Biosciences), anti-KLRG1 (2F1/KLRG1; Biolegend), anti-IFN-γ (XMG1.2; eBioscience), anti-TCRb (H57-597; eBioscience), anti-PD-1 (29F.1A12; Biolgend), anti-CXCR5 (2G8; BD Biosciences) or anti-CXCR5 (L138D7; Biolegend), anti-Ly6C (HK1.4; Biolgend), and anti-GL7 antigen (GL7; Biolegend).

For sorting of naive CD8 T cells, splenocyte samples were initially depleted of B220^+^ cells through the use of magnetic beads (Thermofisher). CD4 T cells were negatively selected using the Dynabeads FlowComp Mouse CD4 Kit (Thermofisher) and then were stained with monoclonal antibodies to purify CD62L^+^ CD44^−^ CD25^−^ cells on a FACSAria II or FACSAriaIII (BD Biosciences). For phenotypic analysis, cells were stained with monoclonal antibodies, phycoerythrin (PE)-conjugated H-2D^b^-gp(33–41) (MBL International) for 1 hour at RT or PE-I-A^b^(gp66-77) (obtained from NIH Tetramer Core) for 90 minutes at 37°C. Dead cells were excluded by staining with DAPI (4,6-diamidino-2-phenylindole; Sigma). Data were analyzed with FlowJo software (TreeStar).

### *In Vitro* T cell culture

Sort purified naive (CD62L^+^ CD44^lo^ CD25^−^) CD4 or CD8 T cells were cultured in RPMI 1640 medium supplemented with 10% FBS (Thermofisher) and Gluta-MAX in the presence of plate-bound anti-CD3 (145-2C11; Biolegend) and soluble anti-CD28 (37.51; Bio X Cell) at concentrations of 0.1 μg/ml and 0.5 μg/ml, respectively, unless specified otherwise, in multiwell tissue culture plates coated with rabbit antibody to hamster IgG (0855395; MP Biomedicals). For T_H_1 polarization 10 ng/mL of IL-12 (R&D Systems) and 10 μg/mL of anti-IL-4 (11B11) were added to culture media. For T_H_2 polarization 10 ng/mL IL-4 (eBioscience), 10 μg/mL anti-IL-12 (BioXCell), and 10 μg/mL anti-IFN-γ (Biolegend) were added to culture media. Following 3 days of anti-CD3 and anti-CD28 stimulation cells were removed from stimulation and rested for 3 days in conditioned medium at concentration of 0.5–1 x 10^6^ cells/mL. For CD8 T cell cultures, cells were cultured in 40 U/mL IL-2 during the resting period.

Day 6 CD4 T cells were harvested, washed in fresh media, and restimulated using 50 ng/mL PMA (Sigma) and 1 μM Ionomycin (Sigma) or plate-bound anti-CD3 (Biolegend) and soluble anti-CD28 (BioXcell) as described above for the indicated times. For intracellular cytokine analysis, Brefeldin A (Biolegend) was added to the cultures 2 hours before harvest. Supernatant was collected 24 hours post-stimulation.

### ELISA

ELISA for cytokines was performed on Nunc Maxisorp plates using ELISA MAX IFN-γ and IL-4 kits (Biolegend) and developed using TMB substrate (Dako). OD_450_ values were read on a spectrophotometer. Standard curves for these ELISAs were generated with purified cytokines.

Anti-LCMV ELISA was performed as previously described [42]. Nunc Polysorp plates were coated with 10ug/mL sonicated cell lysate from LCMV-infected BHK-21 cells (gift from Marco Colonna, Washington University) as capture antigen or uninfected BHK-21 cell lysate overnight followed by UV irradiation (300 mJ in Stratalinker 1800; Stratagene). Plasma antibody was detected with biotinylated anti-mouse IgG1 antibody (BD) and anti-mouse IgG2c antibody (1077–08; Southern Biotech). Endpoint titers were calculated by a sigmoidal-dose response curve using a Graphpad Prism 7 software.

### Intracellular cytokine staining

Cells were subject to LIVE/DEAD Aqua staining (Thermofisher) for 30 minutes at 4°C before being fixed with 4% PFA for 10 minutes at RT. Cells were washed twice with 0.03% saponin (Sigma) in 2% FBS/PBS before being stained with the indicated antibodies in 0.3% Saponin in 2% FBS/PBS for 20 min at 4°C.

### T cell stimulation and western blotting

*In vitro* polarized T cells were rested for 1 hour in cytokine-free RPMI medium before being stained with 5 μg/mL anti-CD3 and 5 μg/mL anti-CD28 on ice for 20 minutes. TCR was then crosslinked by addition of 20 μg/mL of anti-Armenian Hamster IgG to the cells on ice. Cells were stimulated in 5 ml polystyrene tubes for the indicated times in a water bath at 37°C. Cells were lysed in NP-40 Lysis Buffer (1% NP-40, 50 mM Tris-HCl (pH7.5), 150 mM NaCl, 2 mM EDTA, phosphatase inhibitors) and cleared via centrifugation at 14,000 rpm for 10 minutes, then denatured in SDS sample buffer.

Lysates from equal numbers of cells were separated by 8% or 10% SDS PAGE and transferred to nitrocellulose membranes (Bio-Rad), which were incubated with primary antibodies (identified below), followed by detection with horseradish peroxidase–conjugated species-specific antibody to immunoglobulin light chain (115-035-174 or 211-032-171; Jackson ImmunoResearch) and a Luminata HRP substrate (Millipore). The following antibodies were used: anti-CD2AP [10], anti-ERK2 (Santa Cruz, C-14), anti-p-MEK1/2 (Cell Signaling Technology, 9121).

### RNA-sequencing

Total RNA was extracted from ~300,000 sorted cells using manufacturer’s instructions using the RNA XS Kit (Macherey Nagel). cDNA synthesis and amplification were performed with a Pico Input RNA Kit, according to the manufacturer’s instructions (Clontech). Libraries were sequenced on a HiSeq3000 (Illumina) in single-read mode, with a read length of 50 nucleotides producing ~25 million reads per sample. Sequence tags were mapped onto the NCBI37 mm9 with TopHat [43], followed by transcript assembly and Reads Per Kilo base of exon per Million reads (RPKM) estimation using Cufflinks [44–46] on the Galaxy platform (https://usegalaxy.org/). The raw data has been deposited at NCBI GEO with an accession number GSE112778.

### Statistical analyses

Statistical analyses were performed with a two-tailed unpaired Student’s *t*-test or a Mann-Whitney test using GraphPad Prism software.

## Supporting information

S1 Fig CD2AP is dispensable for T cell development. (A) Generation of a *Cd2ap*-flox allele by gene targeting. Arrows indicate primer position for PCR. LA: long homology arm, SA: short homology arm, Neo: neomycin resistance cassette, DTA: diphtheria toxin A. (B, C) Flow cytometric analysis of expression of CD4, CD8, TCR, CD24 and CD69 in thymocytes (B) and splenocytes (C) from *Cd4*-cre^+^ *Cd2ap*^F/F^ and control cre^−^*Cd2ap*^F/F^ mice. Numbers indicates percentages of cells surrounded by rectangle or polygon gates. Data are representative of 3–6 mice in 2 independent experiments.
(TIF)

S2 Fig Time-course of GC B cell response following SRBC and NP-CGG immunization in *Cd2ap* deficient mice. (A, C, E) Flow cytometric analysis of expression of PD-1 and CXCR5 on pre-gated CD4^+^ B220^−^ T cells and GL7 and Fas expression on CD19^+^ B220^+^ B Cells at 6 days (A), 22 days (C) following SRBC immunization, and 10 days following Alum precipitated NP-CGG immunization (E). (B, D, F) Numbers and frequencies of CXCR5^+^ PD-1^+^ T_FH_ and Fas^+^ GL7^+^ GC B cells in the spleen of *Cd4*-cre^+^ *Cd2ap*^F/F^ and control *Cd2ap*^F/F^ mice at 6 days (B), 22 days (D) after immunization with SRBC, and 10 days following Alum precipitated NP-CGG immunization (F). Data are representative of 2 independent experiments shown as means and standard deviation.
(TIF)

S3 Fig Analysis of GC response in CD2AP deficient mice following LCMV-c13 infection. (A) Analysis of viral plaque forming units (PFU) in *Cd2ap*^F/F^ and *Cd4*-cre^+^ *Cd2ap*^F/F^ mice at day 45 of LCMV-c13 infection. (B) Expression of PD-1 and CXCR5 in CD4 T cells at day 22 of LCMV-c13 infection in *Cd2ap*^F/F^ and *Cd4*-cre^+^ *Cd2ap*^F/F^ mice (C-D) Absolute numbers of (C) CD4 T cells, and T_FH_ cells (CXCR5^+^PD-1^+^) and (D) B cells at day 22 of LCMV-c13 infection in *Cd2ap*^F/F^ and *Cd4*-cre^+^ *Cd2ap*^F/F^ mice. (E, F) Frequencies of Foxp3^+^ T_FH_ cells 22 days following LCMV-c13 infection of *Cd2ap*^F/F^ and *Cd4*-cre^+^ *Cd2ap*^F/F^ mice. (G) Anti-LCMV IgG1 antibody titers of plasma from *Cd2ap*^F/F^ and *Cd4*-cre^+^ *Cd2ap*^F/F^ mice 60 days after LCMV-c13 infection. (H) Frequencies of Ki67^+^ T_FH_ cells 22 days following LCMV-c13 infection of *Cd2ap*^F/F^ and *Cd4*-cre^+^ *Cd2ap*^F/F^ mice. (I) Expression of ICOS and OX-40 in Naive CD4 (PD-1^−^ CD44^lo^) CD4 T cells and CXCR5^+^ PD-1^+^ T_FH_ cells 22 days following LCMV-Armstrong infection in *Cd2ap*^F/F^ and *Cd4*-cre^+^ *Cd2ap*^F/F^ mice. Data are representative of 2 independent experiments with n = 3–6 mice per genotype.
(TIF)

S4 Fig Analysis of GC output following LCMV-c13 infection. (A-B) Expression of B220, IgM, IgD, GL7, AA4.1, CD138, and CD24 of splenocytes (A) and bone marrow cells (B) of *Cd2ap*^F/F^ and *Cd4*-cre^+^ *Cd2ap*^F/F^ 60 days after LCMV-c13 infection. (C-D) Absolute numbers of memory B cells in the spleen (C) and plasma cells in the bone marrow (D) in *Cd2ap*^F/F^ and *Cd4*-cre^+^ *Cd2ap*^F/F^ mice 60 days after LCMV-c13 infection. Data are representative of 2 independent experiments shown as means and standard deviation.
(TIF)

S5 Fig CD8 T cell response in CD2AP deficient mice is comparable to control mice following LCMV-c13 infection. (A) Expression of CD8, CD4, CD44 and PD-1 and binding of H-2D^b^(gp33-41) in *Cd2ap*^F/F^ and *Cd4*-cre^+^ *Cd2ap*^F/F^ mice 22 days after LCMV-c13 infection (B) Absolute quantification of (A) (C) Splenocytes from *Cd2ap*^F/F^ and *Cd4*-cre^+^ *Cd2ap*^F/F^ mice 22 days after LCMV-c13 infection were stimulated with gp33-41 peptide and the expression of IFN-γ and TNF-α was analyzed. Data are representative of 2 independent experiments with n = 4–6 mice per genotype.
(TIF)

S6 Fig Intact CD8 and CD4 T cell response to LCMV Armstrong in T cell-specific *Cin85* deficient mice. (A) Expression of IFN-γ, TNF-α, and IL-21 in splenocytes from day 8 LCMV-Armstrong following stimulation with gp61-80 peptide in *Cin85*^F/F^ and CD4-cre^+^ *Cin85*^F/F^ animals (B) Expression of CD4, CD8, and CD44, and binding of H-2D^b^(gp33-41) and H-2D^b^(np396-404) tetramers of splenocytes from *Cin85*^F/F^ and *Cd4*-cre^+^ *Cin85*^F/F^ mice eight days after LCMV-Armstrong infection. (C) Expression of CD19, B220, GL7, and Fas eight days after LCMV-Armstrong infection in *Cin85*^F/F^ and *Cd4*-cre^+^ *Cin85*^F/F^ mice (D, E) Absolute quantification of cell numbers from *Cin85*^F/F^ and *Cd4*-cre^+^ *Cin85*^F/F^ mice. Data are representative of 2 independent experiments of n = 4–6 mice each shown as means and standard deviation.
(TIF)

S7 Fig Delayed clearance of LCMV-c13 in *Vav1*-icre *Cin85*^F/F^ mice. (A) Plasma viral abundance of LCMV determined by *gp* transcripts in *Cin85*^F/F^ and *Vav1*-icre^+^ *Cin85*^F/F^. (B-D) Frequencies and absolute numbers of (B) H-2D^b^(gp33-41)-specific CD8^+^ cells (C) I-A^b^(gp66-77)-specific CD4 T cells and CXCR5^+^ PD-1^+^ CD4 T cells, and (D) Fas^+^ GL7^+^ B cells in the spleen day 30 post-infection in LCMV-c13 infection in *Cin85*^F/F^ and *Vav1*-icre^+^ *Cin85*^F/F^ mice. Data combined from three independent experiments of n = 2–3 mice each.
(TIF)
