# Supplementary figures and images for: The adaptor molecule CD2AP in CD4 T cells modulates differentiation of follicular helper T cells during chronic LCMV infection
Source: PLoS Pathog. 2018 May 7;14(5):e1007053. doi: 10.1371/journal.ppat.1007053 (PMC5957453; doi:10.1371/journal.ppat.1007053)

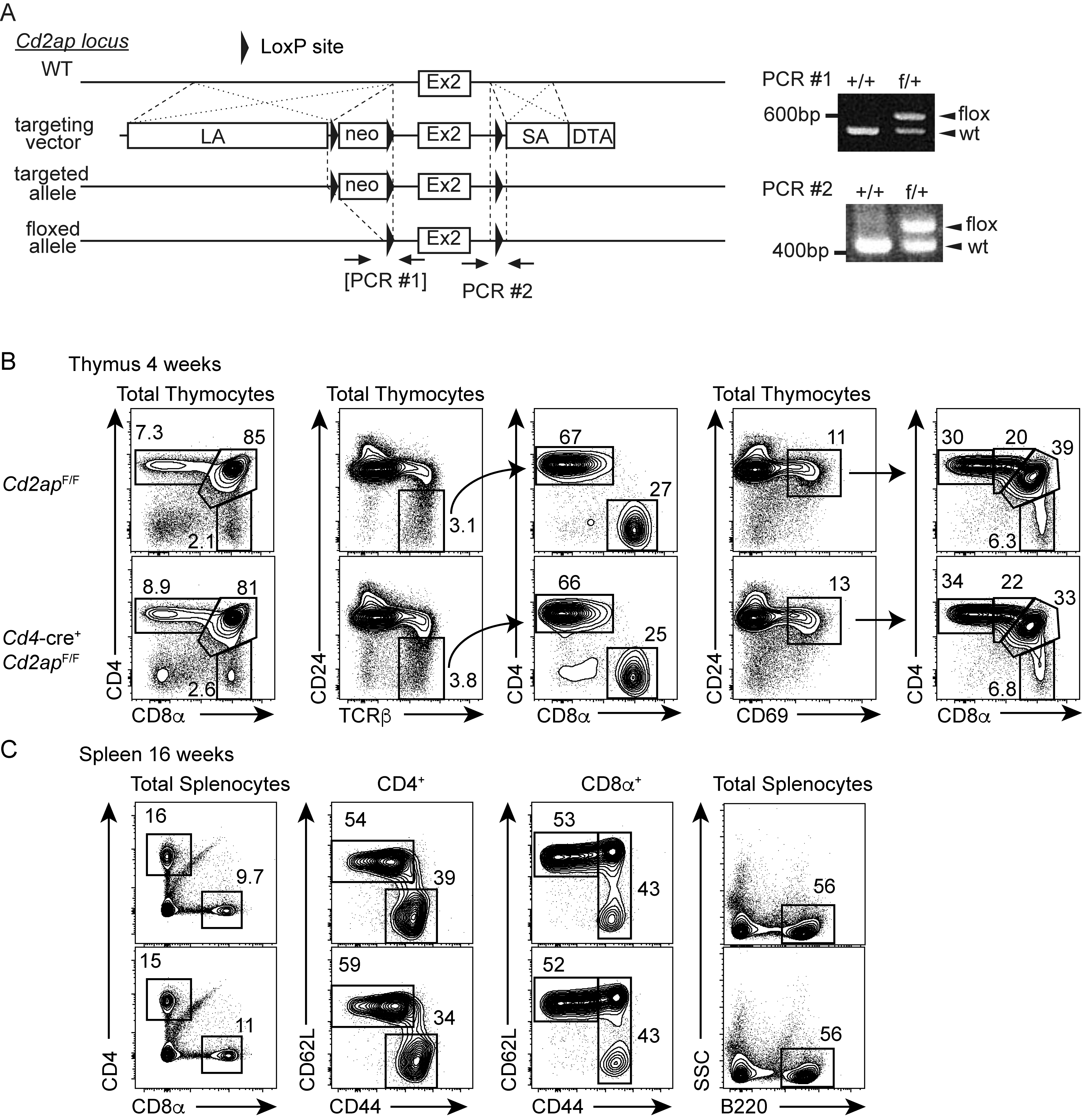

Supplement: S1 Fig — (A) Generation of a Cd2ap-flox allele by gene targeting. Arrows indicate primer position for PCR. LA: long homology arm, SA: short homology arm, Neo: neomycin resistance cassette, DTA: diphtheria toxin A. (B, C) Flow cytometric analysis of expression of CD4, CD8, TCR, CD24 and CD69 in thymocytes (B) and splenocytes (C) from Cd4-cre+ Cd2apF/F and control cre−Cd2apF/F mice. Numbers indicates percentages of cells surrounded by rectangle or polygon gates. Data are representative of 3–6 mice in 2 independent experiments. (TIF) [file ppat.1007053.s001.tif]

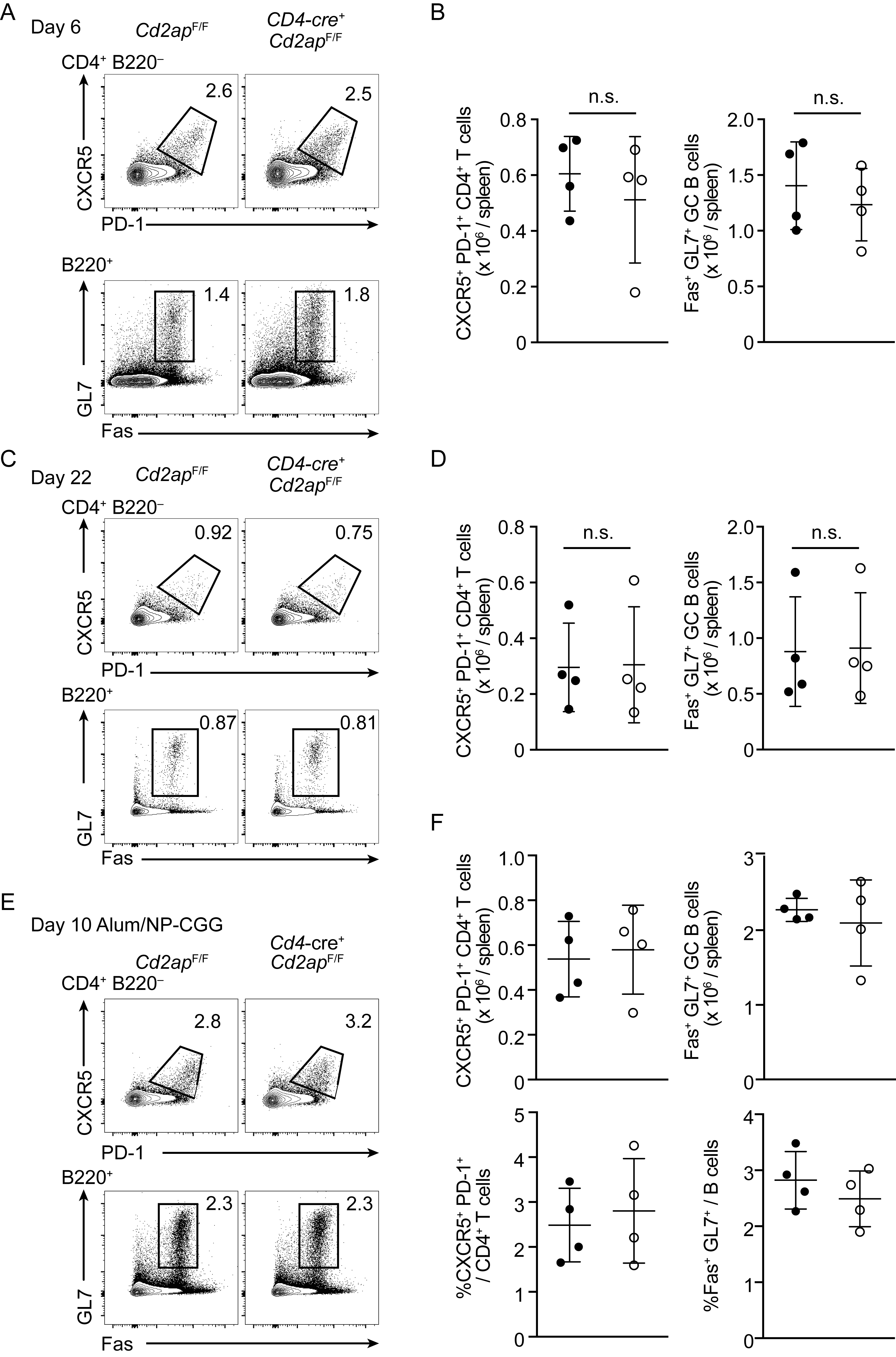

Supplement: S2 Fig — (A, C, E) Flow cytometric analysis of expression of PD-1 and CXCR5 on pre-gated CD4+ B220− T cells and GL7 and Fas expression on CD19+ B220+ B Cells at 6 days (A), 22 days (C) following SRBC immunization, and 10 days following Alum precipitated NP-CGG immunization (E). (B, D, F) Numbers and frequencies of CXCR5+ PD-1+ TFH and Fas+ GL7+ GC B cells in the spleen of Cd4-cre+ Cd2apF/F and control Cd2apF/F mice at 6 days (B), 22 days (D) after immunization with SRBC, and 10 days following Alum precipitated NP-CGG immunization (F). Data are representative of 2 independent experiments shown as means and standard deviation. (TIF) [file ppat.1007053.s002.tif]

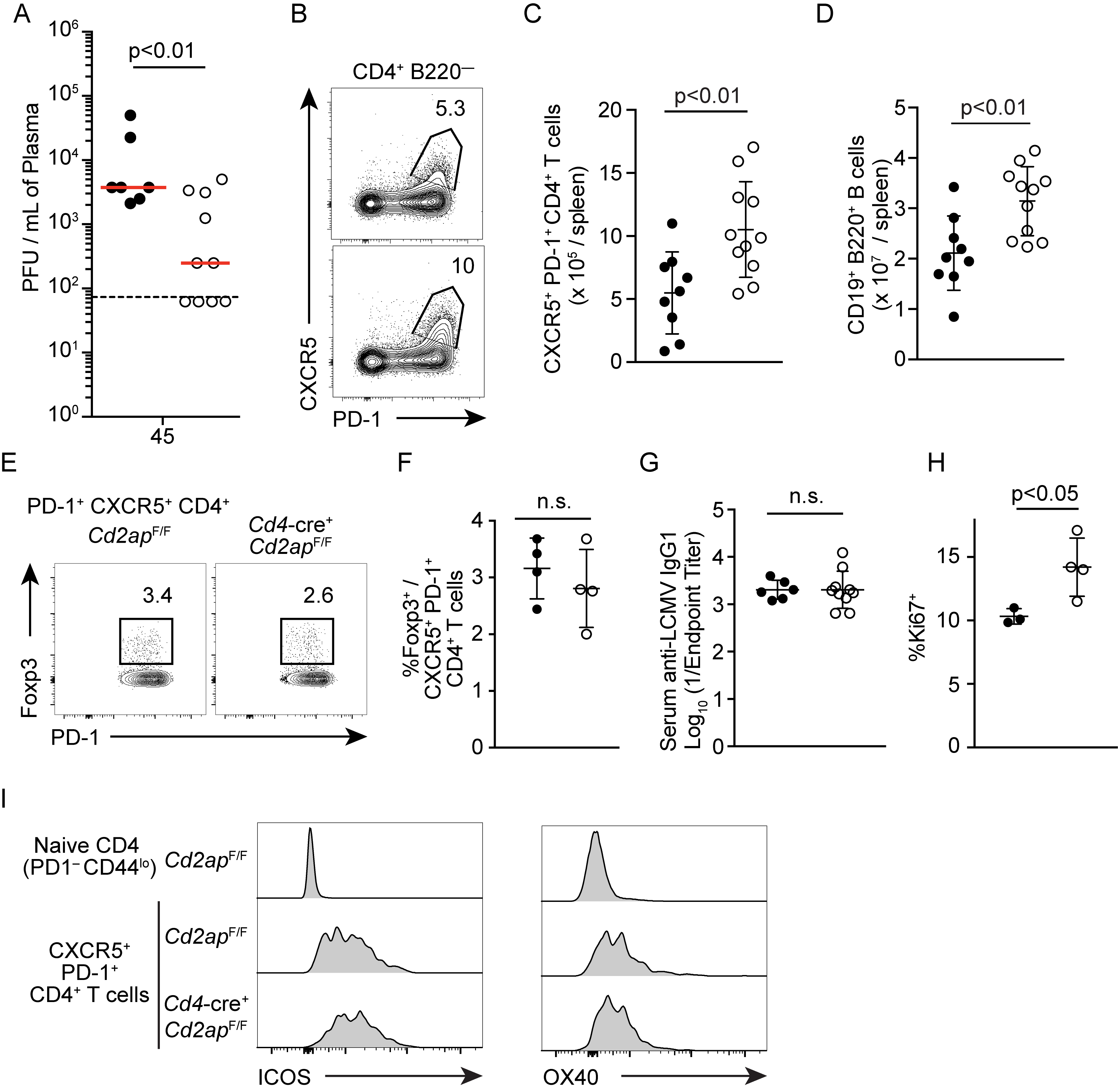

Supplement: S3 Fig — (A) Analysis of viral plaque forming units (PFU) in Cd2apF/F and Cd4-cre+ Cd2apF/F mice at day 45 of LCMV-c13 infection. (B) Expression of PD-1 and CXCR5 in CD4 T cells at day 22 of LCMV-c13 infection in Cd2apF/F and Cd4-cre+ Cd2apF/F mice (C-D) Absolute numbers of (C) CD4 T cells, and TFH cells (CXCR5+PD-1+) and (D) B cells at day 22 of LCMV-c13 infection in Cd2apF/F and Cd4-cre+ Cd2apF/F mice. (E, F) Frequencies of Foxp3+ TFH cells 22 days following LCMV-c13 infection of Cd2apF/F and Cd4-cre+ Cd2apF/F mice. (G) Anti-LCMV IgG1 antibody titers of plasma from Cd2apF/F and Cd4-cre+ Cd2apF/F mice 60 days after LCMV-c13 infection. (H) Frequencies of Ki67+ TFH cells 22 days following LCMV-c13 infection of Cd2apF/F and Cd4-cre+ Cd2apF/F mice. (I) Expression of ICOS and OX-40 in Naive CD4 (PD-1− CD44lo) CD4 T cells and CXCR5+ PD-1+ TFH cells 22 days following LCMV-Armstrong infection in Cd2apF/F and Cd4-cre+ Cd2apF/F mice. Data are representative of 2 independent experiments with n = 3–6 mice per genotype. (TIF) [file ppat.1007053.s003.tif]

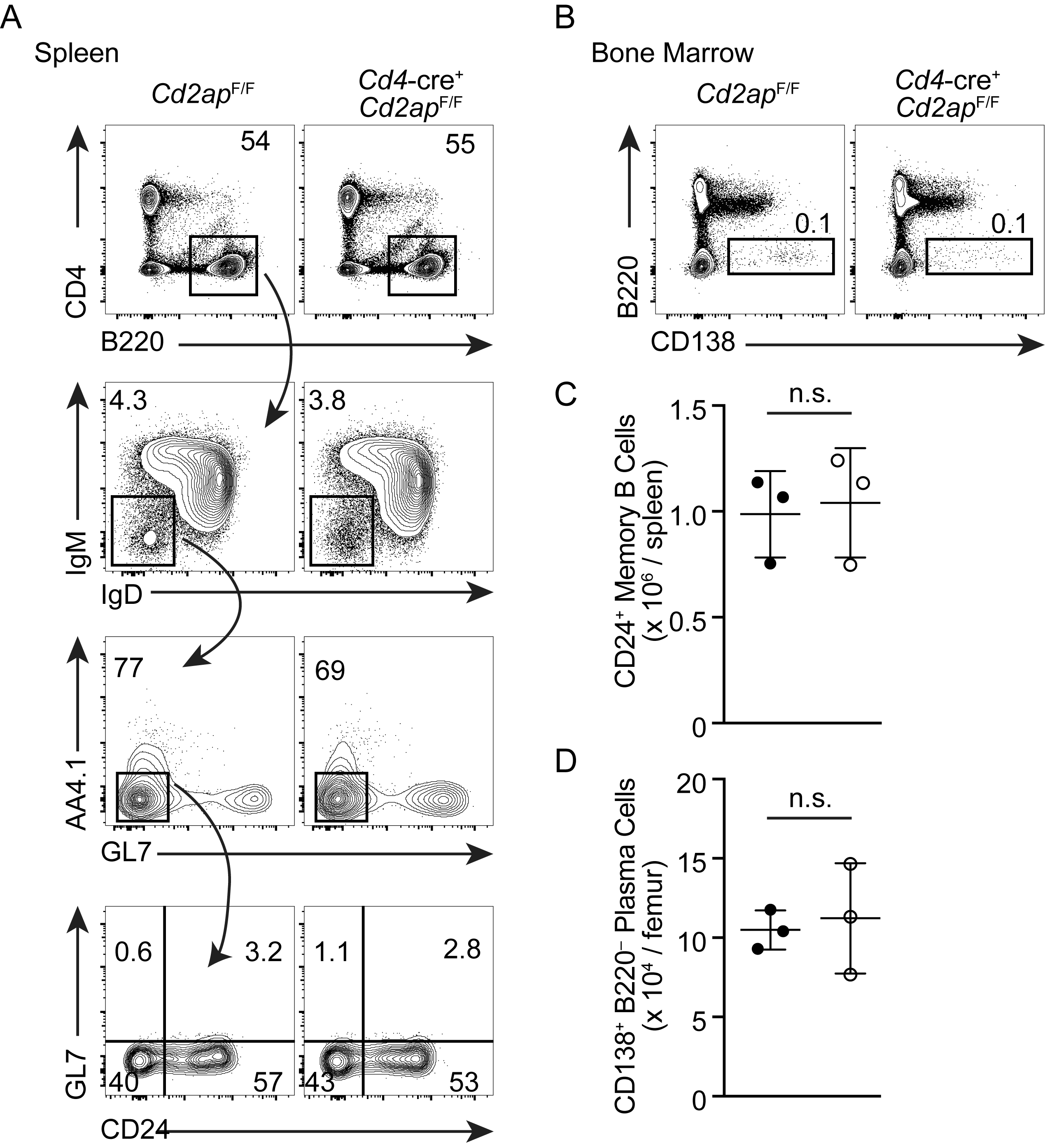

Supplement: S4 Fig — (A-B) Expression of B220, IgM, IgD, GL7, AA4.1, CD138, and CD24 of splenocytes (A) and bone marrow cells (B) of Cd2apF/F and Cd4-cre+ Cd2apF/F 60 days after LCMV-c13 infection. (C-D) Absolute numbers of memory B cells in the spleen (C) and plasma cells in the bone marrow (D) in Cd2apF/F and Cd4-cre+ Cd2apF/F mice 60 days after LCMV-c13 infection. Data are representative of 2 independent experiments shown as means and standard deviation. (TIF) [file ppat.1007053.s004.tif]

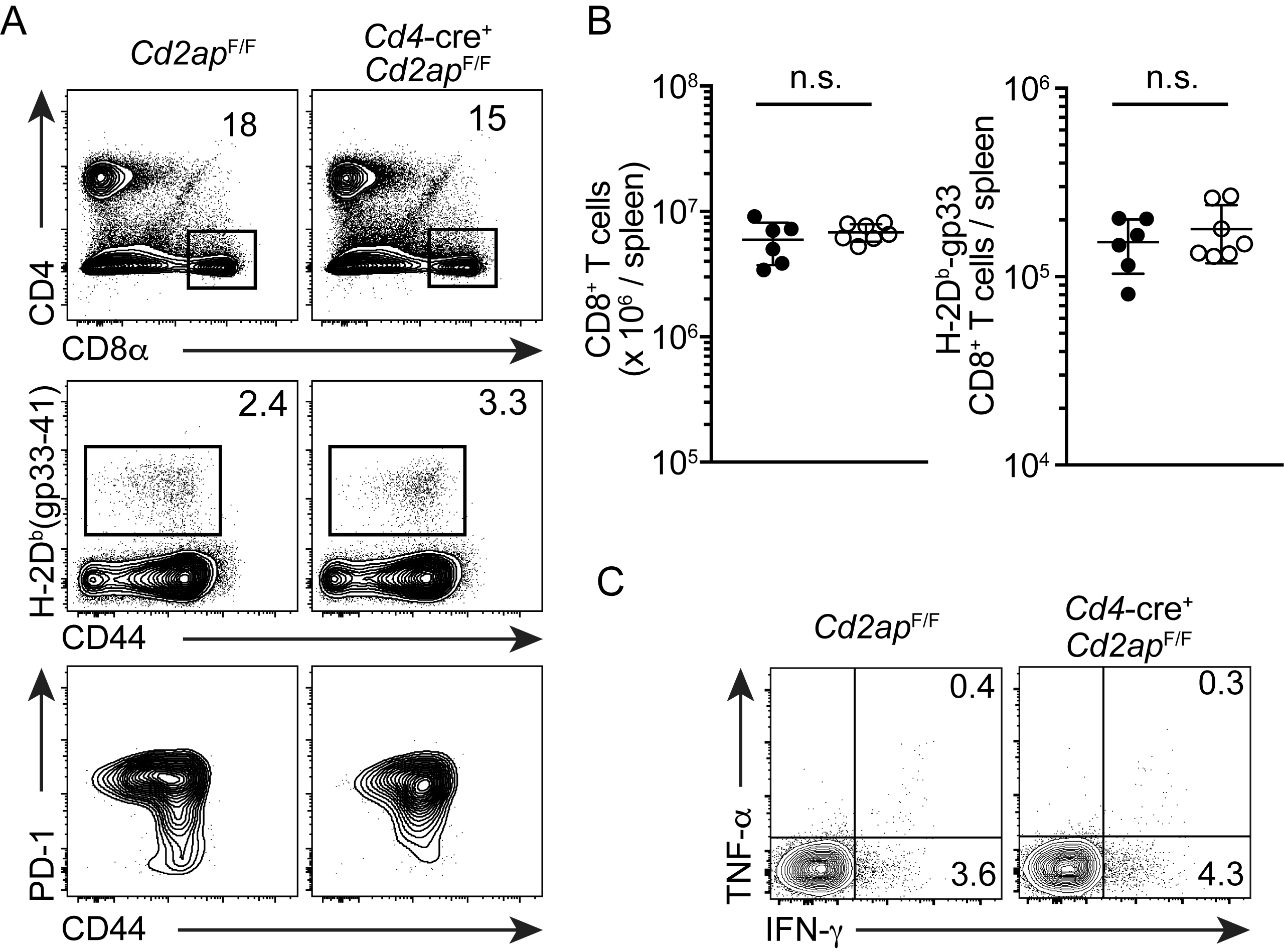

Supplement: S5 Fig — (A) Expression of CD8, CD4, CD44 and PD-1 and binding of H-2Db(gp33-41) in Cd2apF/F and Cd4-cre+ Cd2apF/F mice 22 days after LCMV-c13 infection (B) Absolute quantification of (A) (C) Splenocytes from Cd2apF/F and Cd4-cre+ Cd2apF/F mice 22 days after LCMV-c13 infection were stimulated with gp33-41 peptide and the expression of IFN-γ and TNF-α was analyzed. Data are representative of 2 independent experiments with n = 4–6 mice per genotype. (TIF) [file ppat.1007053.s005.tif]

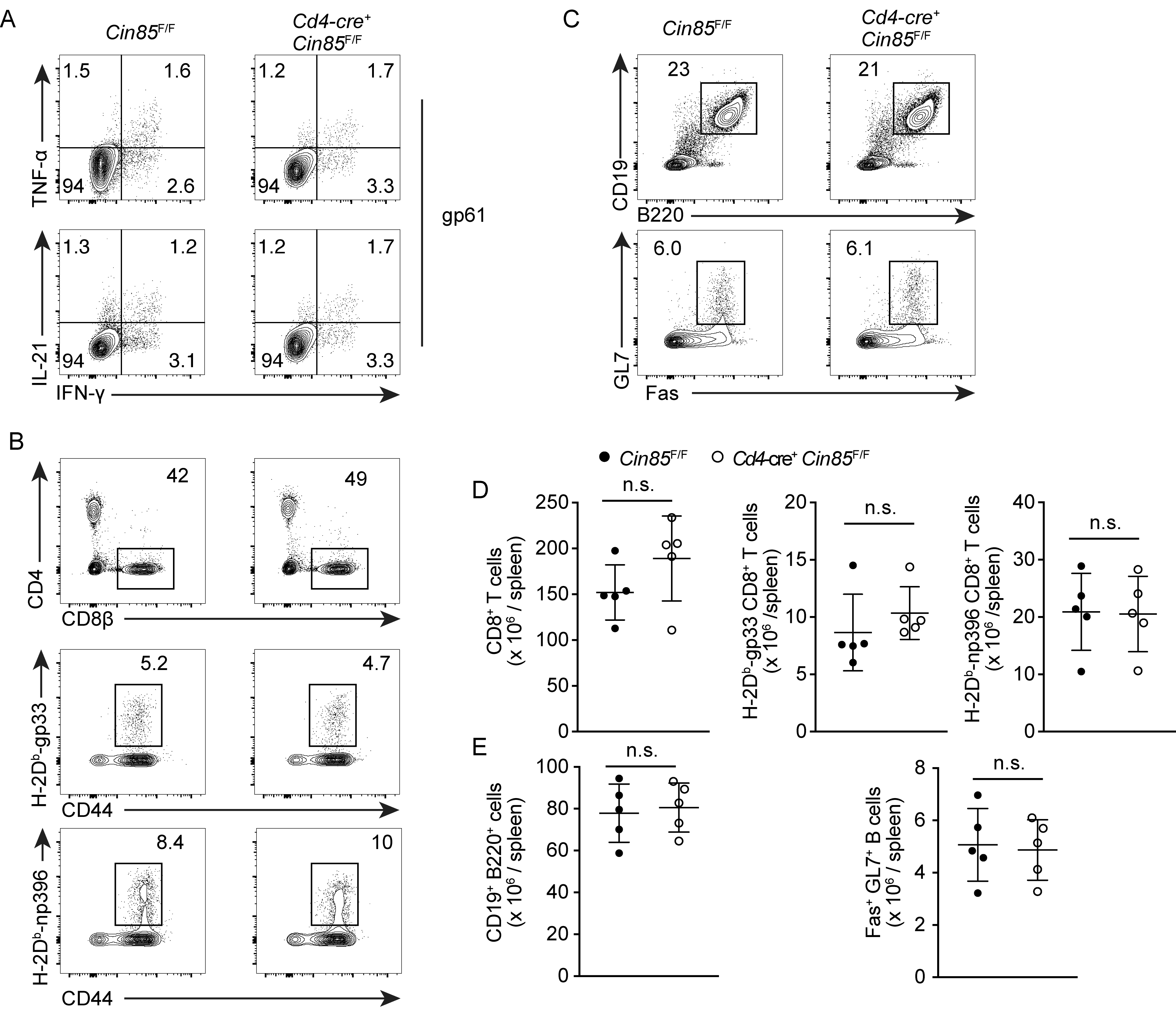

Supplement: S6 Fig — (A) Expression of IFN-γ, TNF-α, and IL-21 in splenocytes from day 8 LCMV-Armstrong following stimulation with gp61-80 peptide in Cin85F/F and CD4-cre+ Cin85F/F animals (B) Expression of CD4, CD8, and CD44, and binding of H-2Db(gp33-41) and H-2Db(np396-404) tetramers of splenocytes from Cin85F/F and Cd4-cre+ Cin85F/F mice eight days after LCMV-Armstrong infection. (C) Expression of CD19, B220, GL7, and Fas eight days after LCMV-Armstrong infection in Cin85F/F and Cd4-cre+ Cin85F/F mice (D, E) Absolute quantification of cell numbers from Cin85F/F and Cd4-cre+ Cin85F/F mice. Data are representative of 2 independent experiments of n = 4–6 mice each shown as means and standard deviation. (TIF) [file ppat.1007053.s006.tif]

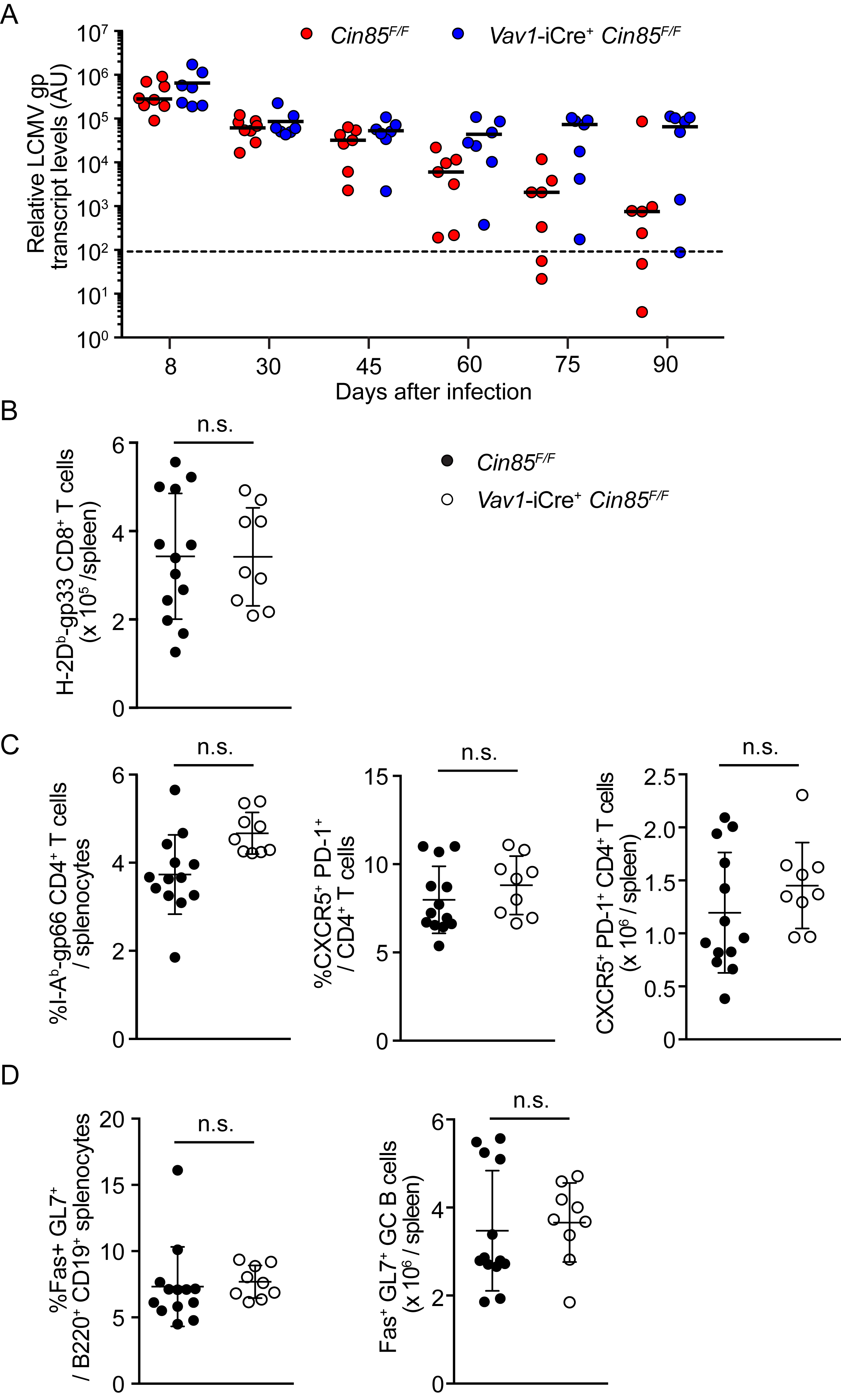

Supplement: S7 Fig — (A) Plasma viral abundance of LCMV determined by gp transcripts in Cin85F/F and Vav1-icre+ Cin85F/F. (B-D) Frequencies and absolute numbers of (B) H-2Db(gp33-41)-specific CD8+ cells (C) I-Ab(gp66-77)-specific CD4 T cells and CXCR5+ PD-1+ CD4 T cells, and (D) Fas+ GL7+ B cells in the spleen day 30 post-infection in LCMV-c13 infection in Cin85F/F and Vav1-icre+ Cin85F/F mice. Data combined from three independent experiments of n = 2–3 mice each. (TIF) [file ppat.1007053.s007.tif]
